# Therapeutic adenine base editor with minimized off-target effects

**DOI:** 10.1093/procel/pwag006

**Published:** 2026-02-05

**Authors:** Yongsen Sun, Nana Yan, Hu Feng, Hongjiang Lu, Zhenrui Zuo, Chikai Zhou, Erwei Zuo

**Affiliations:** State Key Laboratory of Genome and Multi-omics Technologies, Shenzhen Branch, Guangdong Laboratory of Lingnan Modern Agriculture, Key Laboratory of Gene Editing Technologies (Hainan), Ministry of Agriculture and Rural Affairs, Agricultural Genomics Institute at Shenzhen, Chinese Academy of Agricultural Sciences, Shenzhen 518120, China; State Key Laboratory of Genome and Multi-omics Technologies, Shenzhen Branch, Guangdong Laboratory of Lingnan Modern Agriculture, Key Laboratory of Gene Editing Technologies (Hainan), Ministry of Agriculture and Rural Affairs, Agricultural Genomics Institute at Shenzhen, Chinese Academy of Agricultural Sciences, Shenzhen 518120, China; State Key Laboratory of Genome and Multi-omics Technologies, Shenzhen Branch, Guangdong Laboratory of Lingnan Modern Agriculture, Key Laboratory of Gene Editing Technologies (Hainan), Ministry of Agriculture and Rural Affairs, Agricultural Genomics Institute at Shenzhen, Chinese Academy of Agricultural Sciences, Shenzhen 518120, China; State Key Laboratory of Genome and Multi-omics Technologies, Shenzhen Branch, Guangdong Laboratory of Lingnan Modern Agriculture, Key Laboratory of Gene Editing Technologies (Hainan), Ministry of Agriculture and Rural Affairs, Agricultural Genomics Institute at Shenzhen, Chinese Academy of Agricultural Sciences, Shenzhen 518120, China; Key Laboratory of Agricultural Animal Genetics, Breeding and Reproduction, Ministry of Education & College of Animal Science and Technology, Huazhong Agricultural University, Wuhan 430070, China; State Key Laboratory of Genome and Multi-omics Technologies, Shenzhen Branch, Guangdong Laboratory of Lingnan Modern Agriculture, Key Laboratory of Gene Editing Technologies (Hainan), Ministry of Agriculture and Rural Affairs, Agricultural Genomics Institute at Shenzhen, Chinese Academy of Agricultural Sciences, Shenzhen 518120, China; State Key Laboratory of Genome and Multi-omics Technologies, Shenzhen Branch, Guangdong Laboratory of Lingnan Modern Agriculture, Key Laboratory of Gene Editing Technologies (Hainan), Ministry of Agriculture and Rural Affairs, Agricultural Genomics Institute at Shenzhen, Chinese Academy of Agricultural Sciences, Shenzhen 518120, China; State Key Laboratory of Genome and Multi-omics Technologies, Shenzhen Branch, Guangdong Laboratory of Lingnan Modern Agriculture, Key Laboratory of Gene Editing Technologies (Hainan), Ministry of Agriculture and Rural Affairs, Agricultural Genomics Institute at Shenzhen, Chinese Academy of Agricultural Sciences, Shenzhen 518120, China

**Keywords:** adenine base editor, off-target effects, high-fidelity variant, gene therapy

## Abstract

Genome-wide off-target effect poses a safety risk for clinical use of adenine base editor (ABE), among which ABE8e is one of the most efficient. Genome-wide off-target analysis by two-cell embryo injection (GOTI) analysis showed that the rate of genome-wide single-nucleotide variants (SNVs) in ABE8e-edited cells was ∼30-fold higher than that of spontaneous SNVs in control cells, indicating prevalent off-target effects of ABE8e, but no off-target effect for ABE7.10, from which ABE8e was derived. We performed saturation mutagenesis of eight amino acid sites of the deaminase (TadA8e) within ABE8e and obtained ABE8e^Y149V^ that exhibited high editing efficiency without detectable off-target effect. Furthermore, TadA8e^Y149V^ could be fused with other Cas homologs (PAM-relaxed SpRY, hypercompact SaKKH, or IscB) to expand its target range. Finally, ABE8e^Y149V^ editing of hydroxyphenylpyruvate dioxygenase (*Hpd*) gene prevented lethality in hereditary tyrosinemia type I mice. The high efficiency and fidelity of ABE8e^Y149V^ suggest its potential application in ABE-based gene therapies.

## Introduction

Base editors directly install point mutations in genomic DNA and have been widely applied in functional genomics and therapeutic development (Gaudelli et al., 2017; Komor et al., 2016). Approximately 58% of all known disease-associated genetic variants are point mutations, among which 47% of them could in principle be corrected through A-to-G base editing (Rees and Liu, 2018). Therefore, the development of efficient and reliable adenine base editors (ABEs) provides new possibilities for the treatment of many genetic diseases.

Base editor ABE7.10 was an earlier ABE innovation achieved through directed evolution. It contains two deaminases: a native TadA and an evolved TadA variant (TadA*) (Gaudelli et al., 2017). By optimizing codons, modifying nuclear localization signals (NLS), and utilizing directed evolution or phage-assisted evolution of ABE7.10, several variants with further enhanced editing efficiency and targeting range were developed, such as ABEmax (Koblan et al., 2018), miniABEmax (Grünewald et al., 2019b), ABE8s (Gaudelli et al., 2020), and ABE8e (Richter et al., 2020). The ABE8e contains a single TadA* domain with eight additional amino acid mutations (“TadA8e”) and exhibits 3 to 11 times higher activity than ABE7.10 (Richter et al., 2020). The enhanced capability of ABE8e has raised great interest in its potential application for gene therapy and has been used in preclinical animal studies for many diseases, including spinal muscular atrophy (Arbab et al., 2023), inherited cardiac diseases (Lebek et al., 2023; Reichart et al., 2023), heritable monogenic blood disorders such as sickle cell anemia (Newby et al., 2021) and β-thalassemia (Liao et al., 2023), cystic fibrosis (Sun et al., 2024), and familial hypercholesterolemia (Rothgangl et al., 2021).

Despite these advances, a key safety concern remains: off-target deamination at unintended genomic loci. Such off-target effects can be categorized into two distinct classes. The first is sgRNA-dependent off-target editing, which occurs at genomic sites bearing sequence similarity to the intended target site and typically involves off-target Cas9 binding. The second is sgRNA-independent off-target editing, which arises from the intrinsic deaminase activity of the base editor acting on exposed single-stranded DNA regions, independent of guide RNA specificity. Both classes may undermine the therapeutic utility of base editors. sgRNA-independent effects are particularly challenging to detect, as they do not align with predictable Cas9 binding sites. To address this, a genome-wide, unbiased assay called GOTI (genome-wide off-target analysis by two-cell embryo Injection) was developed (Zuo et al., 2019), allowing detection of *de novo* single-nucleotide variants (SNVs) induced by genome editors by comparing edited and unedited blastomeres from the same embryo. Using GOTI, it was shown that the cytosine editor BE3 induces substantial sgRNA-independent SNVs, while ABE7.10 does not (Zuo et al., 2019). However, whether newer, high-efficiency ABEs such as ABE8e and its derivatives also exhibit such off-target effects remains incompletely characterized.

In this study, we systematically compared the activity of multiple ABEs across a library of 102 sgRNA target sites and confirmed that ABE8e exhibits the highest editing efficiency and a broad editing window. However, GOTI-based analysis revealed that ABE8e induces substantial sgRNA-independent off-target SNVs. To address this, we performed saturation mutagenesis at the eight residues of TadA8e and generated 152 single-residue and 6 double-residue variants. Among these, ABE8e^Y149V^ retained high on-target efficiency, showed a narrower editing window, and induced markedly fewer DNA and RNA off-target edits. Comprehensive comparisons revealed that ABE8e^Y149V^ achieves a more favorable balance than previously reported high-fidelity ABE variants, including ABE8e^V106W^, ABE8e^V82G^, ABE8e^K20A/R21A^, and ABE9, by uniquely combining robust editing with minimal off-target activity. Moreover, TadA8e^Y149V^ was compatible with different Cas homologs and enabled efficient correction at multiple disease-relevant loci in human cells. *In vivo*, dual AAV delivery of ABE8e^Y149V^ rescued the lethal phenotype in hereditary tyrosinemia type I (HTI) mice. These results establish ABE8e^Y149V^ as a highly efficient and specific base editor with strong potential for therapeutic applications.

## Results

### ABE8e exhibits high editing efficiency accompanied by a broad editing window

**Figure 1.**
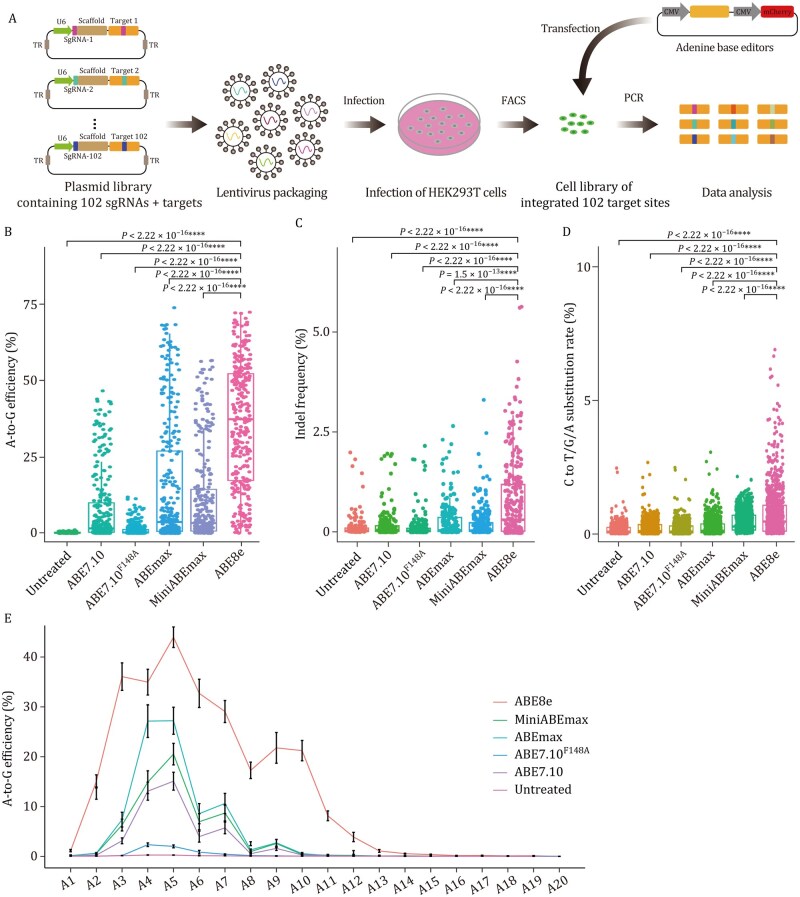
**Comprehensive and unbiased characterization of ABE7.10, ABE7.10^F148A^, ABEmax, miniABEmax and ABE8e in 102-sgRNA cells**. (A) Schematic representation comparing the editing characteristics of different ABEs in HEK293T cells with a stably integrated 102-sgRNA target library. (B) Box plot of the A-to-G editing efficiencies of different ABEs at 102 sgRNA target sites. (C) The proportion of indels in untreated and ABE groups. (D) The proportion of undesired C editing in untreated and ABE groups. The center line in B–D indicates the median, and the bottom and top lines of the box represent the first quartile and third quartile of the values, respectively. Tails extend to the minimum and maximum values. (E) Line graph showing the average A-to-G editing efficiencies of different ABEs at positions A1–A20. “Untreated” refers to cells that were not transfected with any plasmids and served as baseline controls. Data are presented as mean ± SEM of biological replicates (*n *≥ 3, as indicated). *P* values were calculated by two-sided unpaired *t*-test. *P *< 0.05 was considered significant. *P < 0.05. **P < 0.01. ***P < 0.001. ****P < 0.0001.

In order to identify the most suitable ABE-based gene editor for potential clinical application, we first compared the editing efficiency and window size among five well-studied ABEs (ABE7.10, ABE7.10^F148A^, ABEmax, miniABEmax, and ABE8e) (Fig. S1A). To ensure comprehensive and unbiased assessment, we employed an sgRNA-target library detection strategy, as previously reported (Xu et al., 2025). In brief, we constructed a plasmid library comprising 102 sgRNAs paired with corresponding target sequences (Fig. S1B) that were integrated into the genome of HEK293T cells via lentiviral infection (referred to as “102-sgRNA cells” hereafter). These sgRNAs contained at least one adenine located within 1 to 20 nucleotides from the end of the protospacer adjacent motif (PAM; Table S1). Each of the above five ABEs was transfected into 102-sgRNA cells and the base substitution frequencies and indels were subsequently calculated at target sites, respectively (Fig. 1A). Deep sequencing analysis of the 102 target sites in each group of editor-treated cells showed that ABE8e exhibited significantly higher on-target editing efficiency (35.9% on average) than the other ABEs (all ≤15.2%) (Fig. 1B). However, assessment of product purity indicated that ABE8e introduced significantly higher levels of indels and cytosine edits than other ABEs (Fig. 1C and 1D). In addition, using the previously defined criterion that the editing window comprises protospacer positions with ≥30% of the average peak editing efficiency (Arbab et al., 2020; Neugebauer et al., 2023), ABE8e displayed a markedly broader editing window (A2–A10) than the other four ABEs, including ABE7.10 (A4–A7), ABE7.10^F148A^ (A4–A6), ABEmax (A4–A7), and miniABEmax (A3–A7) (Fig. 1E).

### ABE8e introduces substantially more genome-wide off-targets than other ABEs

**Figure 2.**
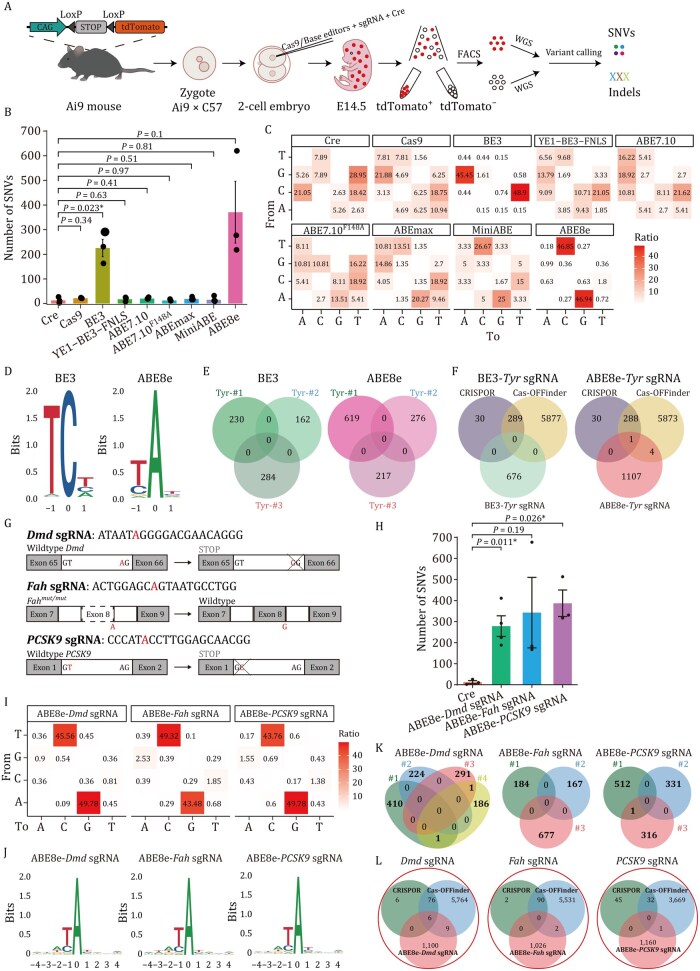
**Unbiased genome-wide off-target analysis of Cas9, BE3, YE1-BE3-FNLS, ABE7.10, ABE7.10^F148A^, ABEmax, miniABEmax and ABE8e**. (A) Schematic diagram of the GOTI method for analyzing the off-target DNA effects of Cas9 and base editors. (B) Comparison of the detected off-target SNVs of Cas9, BE3, YE1-BE3-FNLS, ABE7.10, ABE7.10^F148A^, ABEmax, miniABEmax and ABE8e using an identical *Tyr* sgRNA. (C) Distribution of mutation types for groups injected with Cre, Cas9, two CBEs, and five ABEs. The number indicates the percentage of a certain type of SNVs among all SNVs. Cre samples were derived from our previous studies (Xie et al., 2024; Zuo et al., 2019). (D) Sequence logos derived from off-target DNA SNVs in BE3 and ABE8e groups, respectively. (E) Overlap of off-target SNVs among three BE3-treated embryos and among three ABE8e-treated embryos with identical *Tyr* sgRNA. (F) Overlap among off-target SNVs detected by GOTI with predicted off-targets by Cas-OFFinder and CRISPOR. (G) Schematic showing sgRNA target sites in the *Dmd*, *Fah* and *PCSK9* gene loci. Both *Dmd* and *PCSK9* sgRNAs were targeting exon-intron boundary sequences to inactivate the corresponding genes, altering the AG splice acceptor to GG via editing of A on the sense strand, or altering the GT splice donor to GC via editing of A on the antisense strand. *Fah* sgRNA was targeting *Fah*^−/−^ gene which harbors a G-to-A mutation at the last nucleotide of exon 8 and resulting in exon skipping. (H) Comparison of the detected off-target DNA SNVs for ABE8e with *Dmd*, *Fah* and *PCSK9* sgRNA groups. (I) Distribution of mutation types for ABE8e with *Dmd*, *Fah* and *PCSK9* sgRNA groups. The number indicates the percentage of a certain type of SNVs among all SNVs. (J) Sequence logos derived from off-target sites identified in ABE8e with three sgRNAs. (K) Overlap of off-target SNVs among four ABE8e and *Dmd* sgRNA-treated embryos, three ABE8e and *Fah* sgRNA-treated embryos, and three ABE8e and *PCSK9* sgRNA-treated embryos. (L) Overlap among off-target SNVs detected by GOTI with predicted off-targets by Cas-OFFinder and CRISPOR. Data are presented as mean ± SEM of biological replicates (*n *≥ 3, as indicated). *P* values were calculated by two-sided unpaired *t*-test. *P *< 0.05 was considered significant. *P < 0.05. **P < 0.01. ***P < 0.001. ****P < 0.0001.

To evaluate the genome-wide off-target effects mediated by each of the five ABEs described above, we performed GOTI assays using an sgRNA targeting the *Tyrosinase* (*Tyr*) gene. Given that BE3 reportedly induces substantial genome-wide off-target effects (Jin et al., 2019; Zuo et al., 2019), while YE1-BE3-FNLS shows relatively few such effects (Zuo et al., 2020), we also included Cas9, BE3, and YE1-BE3-FNLS as comparison groups, using the same *Tyr* sgRNA. For this experiment, mRNAs encoding each editor were injected into one blastomere of two-cell embryos derived from Ai9 (CAG-LoxP-Stop-LoxP-tdTomato) mice along with the *Tyr* sgRNA and Cre-encoding mRNAs. On embryonic day 14.5 (E14.5), we sorted edited cells (tdTomato^+^) and nonedited cells (tdTomato^−^) by flow cytometry (Fig. 2A). After validating the on-target efficiency by Sanger sequencing (Fig. S2), we performed whole-genome sequencing (WGS) of both edited and nonedited cells (50× coverage) from all treatment groups (*n *≥ 3 embryos per group), and then called SNVs and indels in edited cells using three independent algorithms (Mutect2, Lofreq, and Strelka), with nonedited cells from the same embryo serving as a reference.

The WGS analysis further identified the respective on-target efficiencies of the Cas9, CBE, and ABE editors (Fig. S3A). We found that the average SNVs in edited cells ranged from 12 to 21 per embryo in the ABE7.10, ABE7.10^F148A^, ABEmax, miniABEmax, YE1-BE3-FNLS, and Cas9 groups, a level not significantly different from that in the Cre-only control cells (averaged 13 SNVs per embryo) (Fig. 2B). In contrast, we found a relatively high number of SNVs in cells edited with BE3 (averaged 225 per embryo), consistent with previous reports (Jin et al., 2019; Zuo et al., 2019). Unexpectedly, our results also showed that ABE8e induced a markedly higher number of off-target SNVs (averaged 371 per embryo) than BE3 using the same *Tyr* sgRNA, and more than 28 times higher than that detected in the Cre-only control. Analysis of the distribution of base conversion types by each editor revealed predominantly (95%) C-to-T and G-to-A off-target conversion for BE3 editing and predominantly (94%) A-to-G and T-to-C base off-target conversions for ABE8e editing (Figs. 2C, S3B and S3C). In addition, sequence logos plots indicated that BE3 generally introduced off-targets in the TC sequence context, whereas ABE8e preferentially induced off-targets at TA sites (Fig. 2D). These biases were the same as those respectively observed with the cytosine deaminase, APOBEC1 (Komor et al., 2016) and adenine deaminase TadA8e (Xiao et al., 2024). Moreover, we found that none of the off-target sites were shared among various ABE8e-treated embryos (Fig. 2E) or overlapped with predicted off-targets of co-injected sgRNA (Fig. 2F), indicating that ABE8e-induced mutations were sgRNA-independent and attributed to TadA8e, similar to the that found for BE3. These TadA8e-induced *de novo* mutations were randomly distributed across the chromosomes (Fig. S3D), and we also noted that all eight of these editors had a slightly higher number of indels in edited cells relative to the Cre control, although none of the average indel counts exceeded 13 per embryo (Fig. S3E). To further validate the genome-wide off-target effects of ABE8e, we included an additional sgRNA that did not target any sequence in either the human or mouse genome, referred to as the nontargeting (NT) sgRNA (Fig. S4). In line with the above findings, both BE3 and ABE8e induced significantly more SNVs compared with the Cre-only control group. Specifically, ABE8e treatment resulted in an average of 313 SNVs per embryo, exceeding the number induced by BE3 (average of 270 SNVs per embryo). In contrast, no significant differences in SNV counts were observed between any of the other editor groups and Cre control. These results suggested that ABE8e introduces substantially more genome-wide off-target SNVs than other ABE variants, and even more than the well-characterized genome-wide off-target effects associated with BE3.

ABEs are frequently applied to introduce or revert point mutations in genes associated with diseases, enabling the creation or study of disease models in mice. To further verify the off-target effects of ABE8e, we conducted GOTI assays using three distinct sgRNAs targeting disease-related genes, including *Dmd* (Liang et al., 2018), *Fah* (Song et al., 2020) and *PCSK9* (Rothgangl et al., 2021) (Fig. 2G). We observed that ABE8e mediated high-average on-target editing efficiency (*Dmd*, 85.8%; *Fah*, 73.6%; *PCSK9*, 91.3%) (Fig. S5A). In agreement with our off-target analysis for the *Tyr* editing, ABE8e induced an average of 279, 343, and 387 SNVs when targeting *Dmd*, *Fah*, and *PCSK9*, respectively, an SNV level 21 to 30 folds higher than that in the Cre-only control embryos (Figs. 2H and S5B), with A-to-G and T-to-C conversion accounting for 91% to 95% of off-target mutations (Figs. 2I and S5C). Of note, although ABEs have been reported to catalyze cytosine conversions (Jeong et al., 2021; Kim et al., 2019), we detected no obvious off-target C-to-T/G/A cytosine deamination activity with any of these editors. All the off-target sites with three distinct editing consistently showed preferential A to G conversion at TA sites (Fig. 2J). Additionally, no significant overlap in off-target SNVs was detected among replicates of each editing (Fig. 2K), nor did these SNVs overlap with predicted off-target mutations based on sgRNAs (Fig. 2L). Similar to *Tyr*, ABE8e editing of *Dmd*, *Fah*, and *PCSK9* induced a slightly higher number of indels compared with the Cre control (Fig. S5D). These results further confirmed that ABE8e introduces genome-wide off-target effects in an sgRNA-independent manner.

### Saturation mutagenesis of TadA8e yields ABE8e variants with narrowed editing windows and high activity

**Figure 3.**
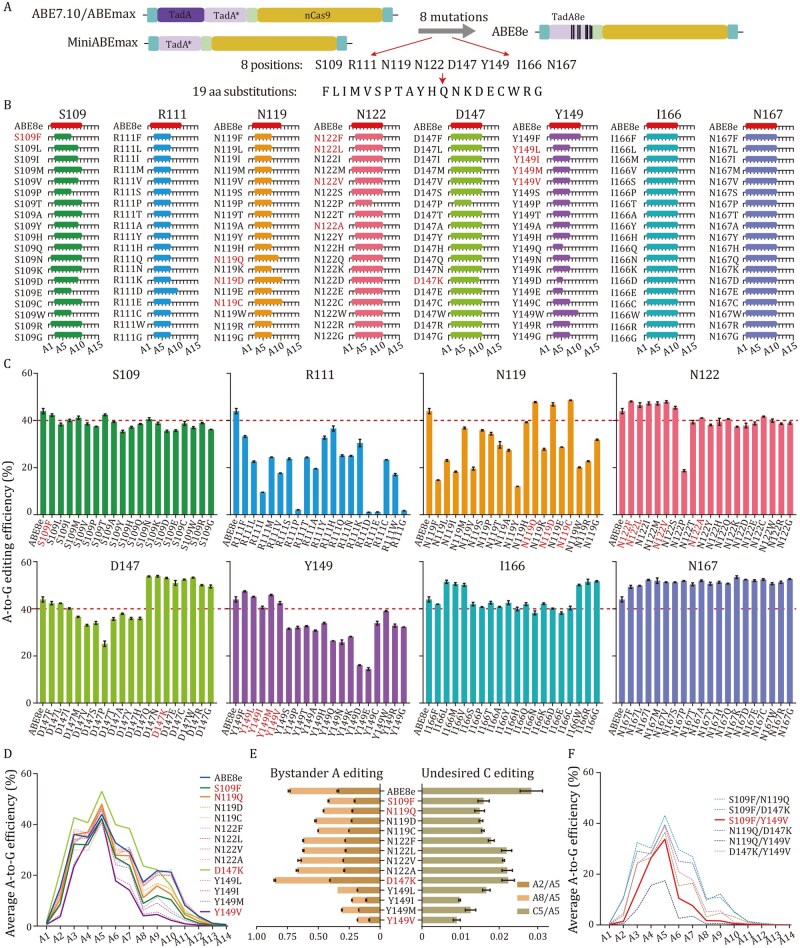
**Re-engineering the eight mutation sites of TadA8e via saturation mutagenesis**. (A) Diagram of saturation mutagenesis at eight sites of ABE8e. (B) The editing windows for ABE8e and each ABE8e variant. Positions edited to equal or more than 30% of the peak editing rate are defined as the editing window. (C) The peak A-to-G editing efficiencies at A5 of ABE8e and its variants. A red dashed line is marked at 40% to distinguish efficiency comparable with (or higher than) ABE8e and efficiency lower than ABE8e. Data are presented as mean ± SEM of three independent biological replicates. (D) Line graph showing the average A-to-G editing efficiencies of 13 variants (S109F, N119Q, N119D, N119C, N122F, N122L, N122V, N122A, D147K, Y149L, Y149I, Y149M and Y149V) at positions A1–A14. (E) Bystander A and undesired C editing of 13 variants. A3 to A7 are defined as within the target window. A2/A5 and A8/A5 represent the ratio of undesired A2 and A8 editing to desired peak A5 editing; C5/A5 represents the ratio of undesired C5 editing to desired A5 editing. Data are presented as mean ± SEM of three independent biological replicates. (F) Line graph showing the average A-to-G editing efficiencies of six double mutants (S109F/N119Q, S109F/D147K, S109F/Y149V, N119Q/D147K, N119Q/Y149V and D147K/Y149V) at A1–A14. Data are derived from three independent experiments.

Previous studies have shown that narrowing the editing windows of deaminases through mutagenesis could reduce sgRNA-independent off-target effects in both DNA and RNA (Chen et al., 2023; Grünewald et al., 2019a; Kim et al., 2017; Zhou et al., 2019; Zuo et al., 2020). We therefore sought to improve ABE8e fidelity by narrowing the editing window of TadA8e. The TadA8e exhibiting high off-target rate was derived from the off-target-free TadA* deaminase in ABE7.10, ABEmax, and miniABEmax, via eight mutations at sites S109, R111, N119, N122, D147, Y149, I166, and N167 (Fig. 3A). We thus surmised that the off-target effects of TadA8e were due to one or more of these mutations.

To narrow the editing window while retaining its high on-target efficiency, we conducted a saturation mutagenesis of these eight sites in TadA8e. Our saturation mutagenesis analysis of single mutations resulted in a panel of 152 ABE8e variants, with 19 different amino acids substituted at each of the eight sites. We then transfected each variant into cell lines harboring 102 sgRNA library and performed targeted deep sequencing analysis. As shown in Figs. 3B, 3C and S6, wild-type ABE8e (A2–A10, peak A-to-G efficiency 44.0%) exhibited a broad editing window, with the highest editing efficiency observed at position A5. Mutations at R111 reduced editing efficiency, whereas mutations at I166 and N167 maintained or increased editing activity. However, alterations at I166 and N167 did not affect the overall width of the editing window. Based on our goal of achieving both higher editing efficiency and a narrower editing window, mutations at R111, I166, and N167 were excluded from further analyses.

Notably, we found that 16 of the 19 mutations at S109 resulted in narrowing of the editing window, with S109F inducing the most pronounced effect (with protospacer positions A3–A7) and showing the highest editing efficiency (averaged 42.3% at A5). All mutations at position N119 narrowed the editing window by one to four nucleotides, with reduced protospacer positions and higher editing efficiency than ABE8e (N119Q, A3–A9, 47.8%; N119D, A3–A10, 46.8%; N119C, A3–A10, 48.6%). Four mutations at N122 (N122F, N122L, N122V, and N122A) exhibited relatively high editing efficiencies (48.1%, 46.6%, 47.9%, and 41.0%, respectively) and slight narrowing of the editing windows (A2–A9). The D147K mutation also induced slight narrowing of the editing window (A2–A9) but significantly higher editing efficiency (53.1%) than ABE8e. The majority of Y149 mutations conferred significant narrowing of the editing windows (A3–A7), and some of which exhibited high editing efficiency (Y149L, 45.1%; Y149I, 40.6%; Y149M, 45.9%; Y149V, 42.4%).

Further comparison of A-to-G editing at different protospacer positions, bystander A editing, and undesired cytosine conversion among the following 13 mutations at 5 different sites: S109F, N119Q, N119D, N119C, N122F, N122L, N122V, N122A, D147K, Y149L, Y149I, Y149M, and Y149V (Fig. 3D and 3E). Among these mutations, Y149V resulted in the narrowest editing window (A3–A7), with the lowest average bystander A editing (A2/A5 + A8/A5: 0.18) and undesired C editing (C5/A5: 0.0090), and was therefore chosen as the best candidate among all single mutations of ABE8e. The S109F and N119Q mutations ranked second and third, respectively, among the single-mutation candidates. Furthermore, we also examined the effects of selectively introducing two simultaneous mutations in the eight amino acid sites. The results showed that combining these mutations generally reduced editing window, but also decreased editing efficiency (Fig. 3F). Notably, the S109F/Y149V combination had a relatively narrower editing window (A3–A6) than S109F and Y149V, but showed lower editing efficiency at A5 (33.8% on average). Based on these results, we selected three ABE8e variants for further investigation: Y149V (42.4% efficiency at A5; window A3–A7, with the lowest bystander and C editing), S109F (42.3% efficiency at A5; window A3–A7, with low bystander and C editing), and the narrower-window variant S109F/Y149V (33.8% efficiency at A5; window A3–A6).

### ABE8e^Y149V^ combines high-efficiency on-target base editing with minimal sgRNA-dependent off-target activity

Several high-precision ABE8e variants have been developed to decrease RNA off-target effects (e.g., ABE8e^V106W^ (Rees et al., 2019; Richter et al., 2020), ABE8e^V82G^ (Grünewald et al., 2019b), ABE8e^K20A/R21A^ (Grünewald et al., 2019b)) or reduce bystander editing (e.g., ABE8e^N108Q/L145T^, also known as ABE9, Chen et al., 2023). To benchmark the newly identified variants, ABE8e^Y149V^, ABE8e^S109F^, and ABE8e^S109F/Y149V^, we first performed a side-by-side comparison with these four previously reported variants in the 102-sgRNA cell library.

**Figure 4.**
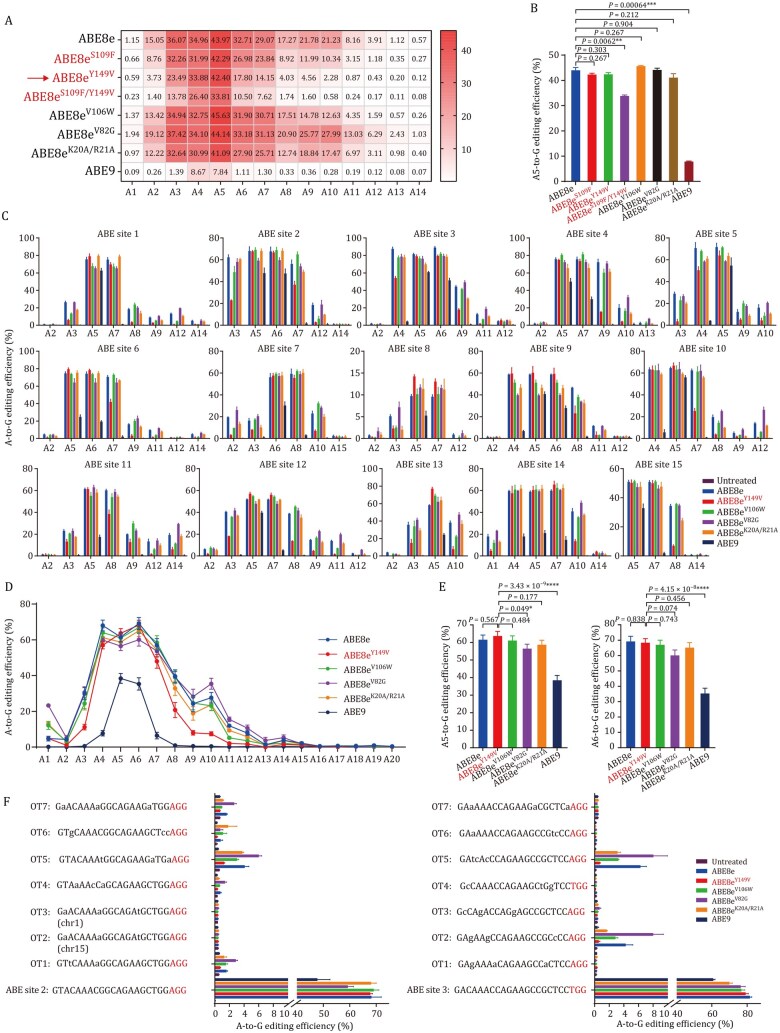
**Comparative evaluation of ABE8e^Y149V^ with wild-type ABE8e and previously reported high-precision ABE8e variants**. (A) Heat map showing the average A-to-G editing efficiencies of ABE8e, three selected variants in this study (ABE8e^S109F^, ABE8e^Y149V^, ABE8e^S109F/Y149V^) and previously reported high-fidelity variants (ABE8e^V106W^, ABE8e^V82G^, ABE8e^K20A/R21A^ and ABE9) at A1–A14 in the 102-sgRNA library. (B) A5 editing efficiencies of eight ABEs in the 102-sgRNA library. (C) The editing efficiency of ABE8e^Y149V^ in comparison with ABE8e and other reported high-fidelity variants across 15 endogenous sites. (D) Line graph showing the average A-to-G editing efficiencies of ABE8e^Y149V^, ABE8e and other reported high-fidelity variants at A1–A20 across 15 endogenous sites. (E) A5 and A6 editing efficiencies across 15 endogenous sites. (F) sgRNA-dependent off-target effects of ABE8e^Y149V^, ABE8e and other reported high-fidelity variants at 14 predicted off-target sites from two loci. Data are presented as mean ± SEM of three independent biological replicates. *P* values were calculated by two-sided unpaired *t*-test. *P *< 0.05 was considered significant. *P < 0.05. **P < 0.01. ***P < 0.001. ****P < 0.0001.

We found that ABE8e^Y149V^ and ABE8e^S109F^ exhibited A5 editing efficiency comparable with those of ABE8e^V106W^, ABE8e^V82G^, and ABE8e^K20A/R21A^; none of which showed a significant decrease relative to wild-type ABE8e. However, ABE8e^Y149V^ displayed a narrower editing window than ABE8e^S109F^, wild-type ABE8e, and the other three variants (Fig. 4A and 4B). Although the dual mutant ABE8e^S109F/Y149V^ exhibited an even narrower editing window than ABE8e^Y149V^, its A5 editing efficiency was reduced by 8.6%. Among all eight ABEs tested, ABE9 showed the lowest overall editing activity, with A4 or A5 editing efficiencies below 9%, which was significantly lower than those of the other variants. Considering this balance between efficiency and precision, ABE8e^Y149V^ was selected for further validation at endogenous genomic loci.

We next evaluated the editing performance of ABE8e^Y149V^ at 15 endogenous sites for comparison with ABE8e and other reported high-fidelity variants (Fig. 4C–E). At key positions A5 and A6, ABE8e^Y149V^ achieved high editing efficiencies (63.7% and 68.3%, respectively), comparable with ABE8e (61.6%, 69.2%), ABE8e^V106W^ (61.1%, 67.0%), ABE8e^V82G^ (56.5%, 60.1%), and ABE8e^K20A/R21A^ (58.7%, 65.1%). However, its editing window (A4–A8) was clearly narrower than those of the above four variants (typically A3–A10). Although ABE9 has a narrower editing window (A5–A6) than ABE8e^Y149V^, its editing efficiencies at the A5 and A6 positions (38.4% and 35.3%) were significantly lower than those of ABE8e^Y149V^.

To further assess sgRNA-dependent off-target effects, we examined 14 potential off-target sites predicted by Cas-OFFinder, associated with two sgRNA target loci (ABE site 2 and ABE site 3) (Fig. 4F). ABE8e^V82G^ showed higher off-target activity than ABE8e (maximum efficiency: 8.0% vs. 6.3%), while ABE8e^V106W^ and ABE8e^K20A/R21A^ induced moderately lower off-target effects. ABE8e^Y149V^ displayed very low off-target activity, comparable with ABE9 at most sites, with a maximum efficiency not exceeding 1.3%.

Taken together, comprehensive comparisons across the 102-sgRNA cell library and multiple endogenous loci revealed that ABE8e^Y149V^ consistently achieved high on-target efficiency while minimizing sgRNA-dependent off-target effects.

### ABE8e^Y149V^ minimizes genome-wide and transcriptome-wide off-target effects

Building on its favorable editing characteristics of ABE8e^Y149V^, including high on-target activity, a narrow editing window, and negligible sgRNA-dependent off-target effects, we next sought to determine whether it also exhibits reduced sgRNA-independent off-target activity at both the genomic and transcriptomic levels. To this end, we performed GOTI analysis in mouse embryos and RNA-seq in HEK293T cells, comparing ABE8e^Y149V^ with four other high-fidelity ABE8e variants (ABE8e^V106W^, ABE8e^V82G^, ABE8e^K20A/R21A^, and ABE9).

**Figure 5.**
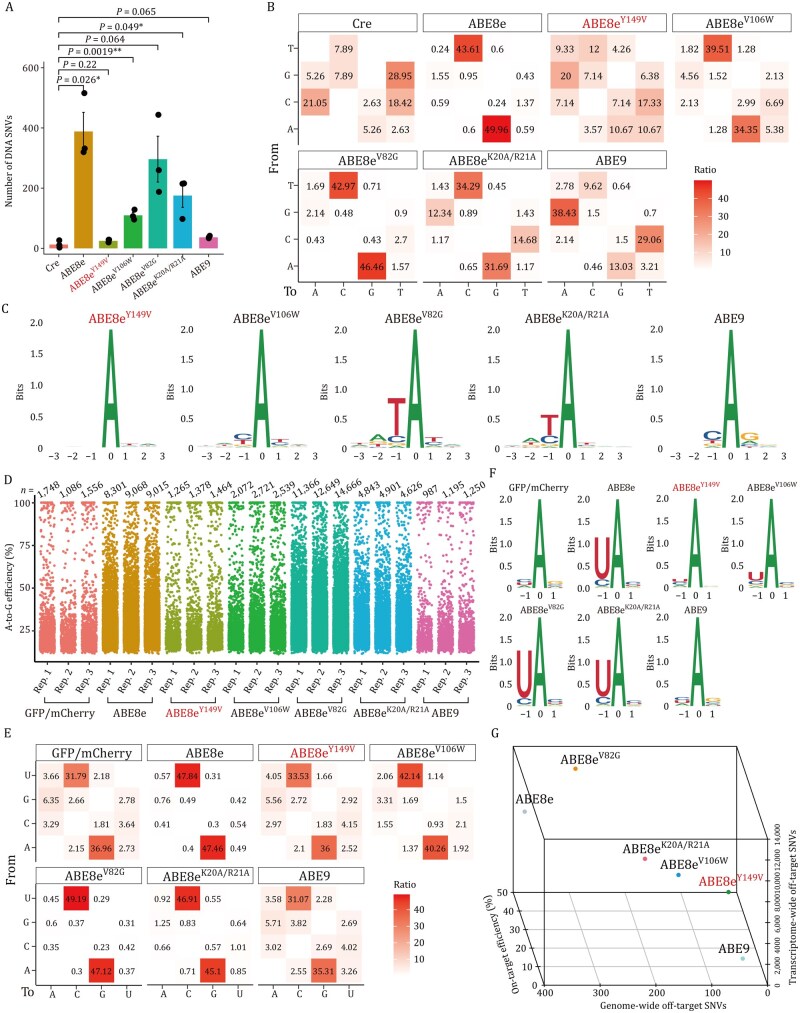
**Genome-wide and transcriptome-wide off-target analysis of ABE8e, ABE8e^Y149V^ and other ABE8e variants**. (A) Comparison of the detected off-target DNA SNVs in Cre, ABE8e, ABE8e^Y149V^ and ABE8e^V106W^, ABE8e^V82G^, ABE8e^K20A/R21A^, and ABE9. (B) Distribution of DNA mutation types for Cre, ABE8e, ABE8e^Y149V^ and other ABE8e variant groups. (C) Sequence logos derived from off-target DNA SNVs in different ABE8e variant groups. (D) Jitter plots showing transcriptome-wide RNA A-to-G edits observed in groups including a GFP/mCherry negative control, ABE8e, ABE8e^Y149V^, and other ABE8e variants. Each dot represents an individual edited adenine. All experiments (except for the GFP/mCherry control) were performed with co-expression of an sgRNA targeting HEK293-site 1, and in all experiments the cells were sorted for the top 5% of GFP and mCherry signals. (E) Distribution of RNA mutation types in GFP/mCherry, ABE8e, ABE8e^Y149V^, and other ABE8e variant groups. (F) Sequence logos derived from off-target RNA SNVs. (G) 3D scatter plot comparing on-target editing efficiencies, genome-wide off-target SNV counts, and transcriptome-wide off-target SNV counts of ABE8e and different ABE8e variants. Data are presented as mean ± SEM of three independent biological replicates. *P* values were calculated by two-sided unpaired *t*-test. *P *< 0.05 was considered significant. *P < 0.05. **P < 0.01. ***P < 0.001. ****P < 0.0001.

For GOTI-based evaluation of genome-wide DNA off-target effects, we used the well-characterized *PCSK9* sgRNA, which has been previously applied for cholesterol reduction in mice and primates (Fig. 2G). Except for ABE9, all variants exhibited high on-target editing efficiencies at the *PCSK9* site in tdTomato^+^ cells (>80%) (Fig. S7A). ABE8e^Y149V^ and the other four variants induced fewer genome-wide SNVs than ABE8e to varying degrees (Fig. 5A). ABE8e^Y149V^ induced the fewest SNVs (24 per embryo on average), whereas ABE8e^V82G^, ABE8e^K20A/R21A^, ABE8e^V106W^, and ABE9 caused relatively high SNV counts (296, 175, 110, and 37 per embryo, respectively). Analysis of SNV mutation patterns revealed predominant A-to-G and T-to-C conversions with a preference for TA motifs in ABE8e^V106W^ (73.9%), ABE8e^V82G^ (89.4%), and ABE8e^K20A/R21A^ (66.0%) groups (Figs. 5B, 5C and S7B). To further validate the low genome-wide DNA off-target activity of ABE8e^Y149V^, we performed an additional GOTI analysis targeting the *Tyr* locus. Consistent with the *PCSK9* results, ABE8e^Y149V^ induced only background-level genome-wide SNVs comparable with the Cre control, with no detectable enrichment of A-to-G/T-to-C substitutions or TA motifs (Fig. S8A–C).

To assess transcriptome-wide RNA off-target effects, we transfected HEK293T cells with each ABE8e variant and an sgRNA targeting HEK293-site 1 (Zhou et al., 2019; Zuo et al., 2020). Cells transfected with GFP/mCherry backbone served as vector-only control group. RNA-seq analysis showed that ABE8e introduced substantially more RNA SNVs (average: 8,795) than the vector control (average: 1,463). Among the variants, ABE8e^V82G^ caused the highest number of RNA SNVs (12,894), followed by ABE8e^K20A/R21A^ (4,790) and ABE8e^V106W^ (2,444), all of which were significantly higher than baseline (Fig. 5D). In contrast, ABE8e^Y149V^ and ABE9 showed no significant increase in RNA SNV counts compared with vector control. Motif and substitution pattern analysis further revealed that 96.3%, 92.0%, and 82.4% of SNVs in ABE8e^V82G^, ABE8e^K20A/R21A^, and ABE8e^V106W^, respectively, were A-to-G or U-to-C transitions, primarily occurring at UA motifs (Fig. 5E and 5F). In contrast, ABE8e^Y149V^ and ABE9 exhibited no apparent mutation bias or motif preference relative to controls. To further confirm the minimal transcriptome-wide RNA off-target effects of ABE8e^Y149V^, we conducted RNA-seq analyses at two additional on-target sites in HEK293T cells (HEK293-site 2 and HEK293-site 3) (Zhou et al., 2019). At both sites, ABE8e^Y149V^ exhibited RNA SNV profiles indistinguishable from the vector control, with no predominant A-to-G or U-to-C conversions and no enrichment of UA motifs (Fig. S8D–F).

Collectively, these results demonstrate that ABE8e^Y149V^ induces the lowest levels of sgRNA-independent off-target SNVs among all tested variants, achieving background-level off-target effects at both genome-wide and transcriptome-wide levels. This favorable balance between activity and fidelity is further illustrated in the 3D scatter plot (Fig. 5G), which summarizes the relationship among on-target efficiency, genome-wide DNA SNVs, and transcriptome-wide RNA SNVs for each variant. These findings establish ABE8e^Y149V^ as an optimized base editor that combines both high precision and robust activity.

### Compatibility of TadA8e^Y149V^ with diverse CRISPR editing systems and expanded PAM accessibility for high-precision base editing

**Figure 6.**
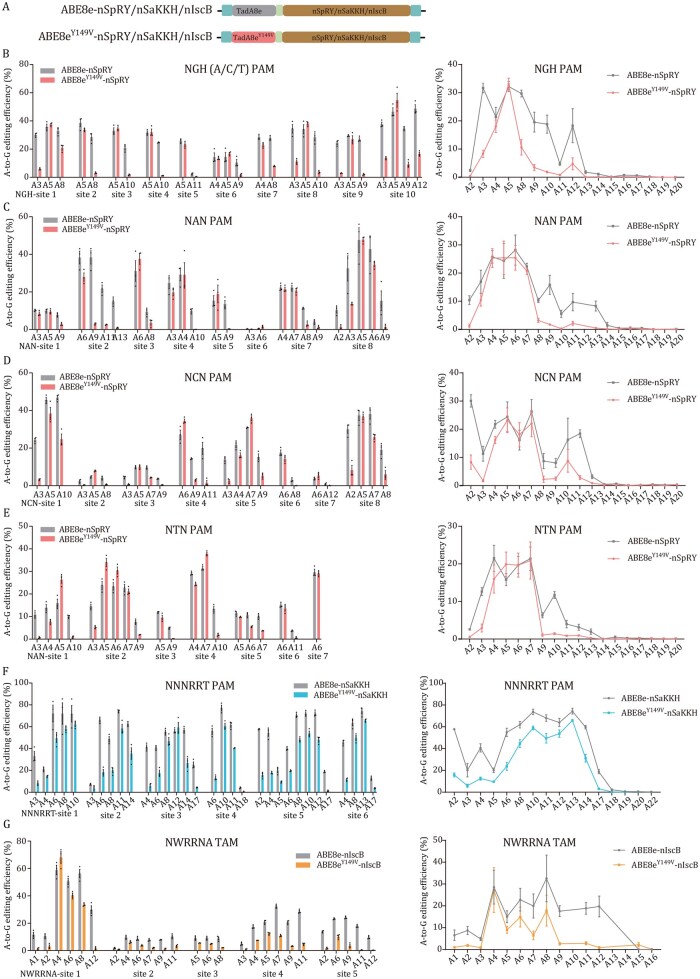
**Characterization of TadA8e^Y149V^ with diverse CRISPR editing systems**. (A) Schematic diagrams of TadA8e and TadA8e^Y149V^ fused with different CRISPR systems. (B–E) A-to-G editing efficiencies of the ABE8e-nSpRY and ABE8e^Y149V^-nSpRY at 33 endogenous loci in HEK293T cells with NGH(A/C/T) (B), NAN(C), NCN(D), or NTN(E) PAMs. Line graph shows the average A-to-G editing efficiencies across positions A1–A20 for both editors with each PAM type. (F) A-to-G editing efficiencies of ABE8e-nSaKKH and ABE8e^Y149V^-nSaKKH at six loci with NNNRRT PAMs. (G) A-to-G editing efficiencies of ABE8e-nIscB and ABE8e^Y149V^-nIscB at five loci with NWRRNA PAMs. Data are presented as mean ± SEM of three independent biological replicates.

Considering the high editing efficiency with negligible off-target effects of ABE8e^Y149V^, we fused TadA8e^Y149V^ with several other CRISPR systems (Fig. 6A) and examined whether its editing characteristics could be preserved and its activity be extended to A sites that were inaccessible via its canonical NGG PAM. Since SpRY nuclease can target al.ost all PAMs (Walton et al., 2020), we replaced nSpCas9 with nSpRY in ABE8e and ABE8e^Y149V^ to construct the ABE8e-nSpRY and ABE8e^Y149V^-nSpRY base editors, respectively. Both editors were then tested at 10, 8, 8, and 7 endogenous loci in HEK293T cells harboring NGH(A/C/T), NAN, NCN, and NTN PAMs, respectively. Targeted deep sequencing revealed that ABE8e^Y149V^-nSpRY achieved comparable A-to-G editing efficiencies with ABE8e-nSpRY at positions A4 to A7 in 9 out of 10 NGH, 7 out of 8 NAN, 6 out of 8 NCN, and 5 out of 7 NTN sites (Fig. 6B–E). This analysis across four PAM types demonstrated that ABE8e^Y149V^-nSpRY maintained a peak editing efficiency comparable with that of ABE8e-nSpRY while exhibiting a noticeably narrower editing window.

In addition, we constructed the ABE8e-nSaKKH and ABE8e^Y149V^-nSaKKH editors to compare their activity at sites with the NNNRRT PAM (Kleinstiver et al., 2015). Tests at six genomic loci indicated that ABE8e-nSaKKH exhibited high efficiency (>50%) between positions A6 to A14. ABE8e^Y149V^-nSaKKH showed high A-to-G substitution activity at positions A10 to A13, with efficiency ranging from 49.5% to 65.8%, slightly lower than that of ABE8e-nSaKKH (Fig. 6F). Furthermore, ABE8e^Y149V^-nSaKKH also demonstrated a narrower editing window (typically from A6 to A14) compared with ABE8e-nSaKKH (from A2 to A17; Fig. 6F).

We also generated the ABE8e-enIscB and ABE8e^Y149V^-enIscB miniature base editors by fusing TadA8e^Y149V^ to the Cas9 ancestor, IscB (496 amino acids), with an NWRRNA TAM (Altae-Tran et al., 2021; Han et al., 2023). Across five genomic loci, ABE8e^Y149V^-enIscB displayed its highest A-to-G conversion efficiency at A4 (27.2% on average), which was comparable with that of ABE8e-enIscB (28.6% on average). However, it showed relatively lower A editing activity at other positions than ABE8e-enIscB (Fig. 6G). Together, these results indicated that TadA8e^Y149V^ was compatible with a PAM-relaxed Cas9 variant, a hypercompact CRISPR, as well as an ancestral Cas9 system, thus expanding the scope of potential targets for high-efficiency adenine base editing.

### Application of ABE8e^Y149V^ at disease-relevant loci in human cells

To explore the therapeutic potential of ABE8e^Y149V^ in correcting disease-associated mutations, we evaluated its on-target editing efficiency at seven clinically relevant loci in human cells using targeted deep sequencing.

**Figure 7.**
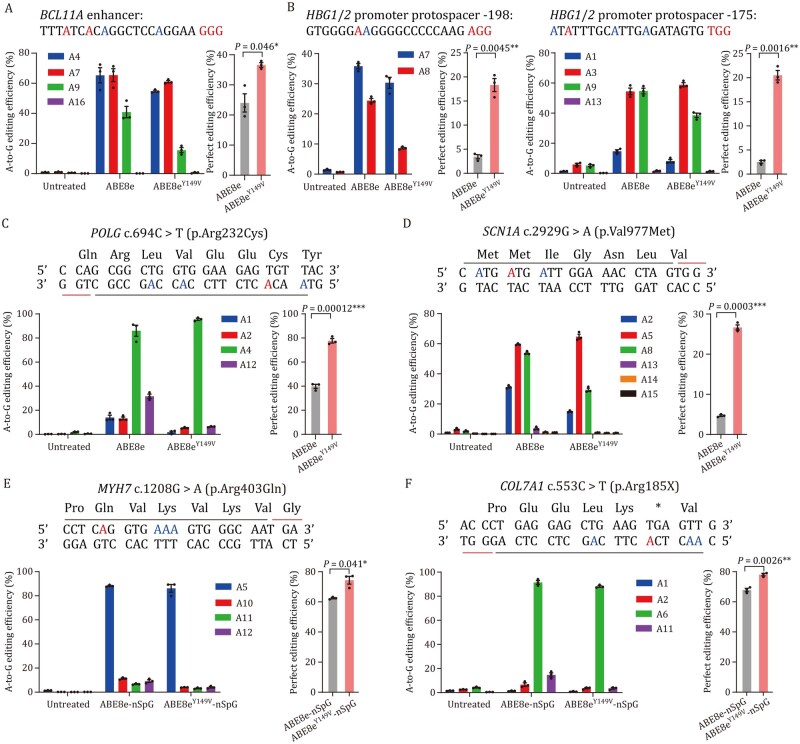
**Application of ABE8e^Y149V^ at disease-relevant loci in human cells**. (AB) Comparison of correcting disease-associated mutations in *BCL11A* enhancer (A) and *HBG1*/*2* promoter (B) induced by ABE8e and ABE8e^Y149V^ in HEK293T cells. (C–F) Comparison of correcting pathogenic mutations induced by ABE8e and ABE8e^Y149V^ in four stable HEK293T cell lines, including *POLG* c.694C>T (C), *SCN1A* c.2929G>A (D), *MYH7* c.1208G>A (E), and *COL7A1* c.553C>T (F). Perfect editing efficiency represents the percentage of deep sequencing reads with precise target-site A-to-G conversion, without bystander edits or indels. Data are presented as mean ± SEM of three independent biological replicates. *P* values were calculated by two-sided unpaired *t*-test. *P *< 0.05 was considered significant. *P < 0.05. **P < 0.01. ***P < 0.001. ****P < 0.0001.

We first targeted the *BCL11A* enhancer, where specific A-to-G conversions are known to upregulate fetal hemoglobin expression, a well-established therapeutic approach for treating hemoglobinopathies such as sickle cell disease and β-thalassemia (Richter et al., 2020). In HEK293T cells, we compared the ability of ABE8e and ABE8e^Y149V^ to simultaneously edit A4 and A7 within a single protospacer. Although ABE8e^Y149V^ exhibited slightly lower editing efficiency at A4 (54.8% vs. 65.3%) and A7 (61.1% vs. 65.4%), likely due to these positions being near the edge of its narrowed editing window, it generated a significantly higher proportion of reads with dual edits at both positions, consistent with its improved precision (Fig. 7A).

We next targeted two clinically relevant variants in the *HBG1* and *HBG2* promoter regions at positions −198 (A7) and −175 (A3), which are also known to enhance fetal hemoglobin expression (Mayuranathan et al., 2023; Richter et al., 2020). At position −198, ABE8e^Y149V^ exhibited slightly lower editing efficiency compared to ABE8e (30.3% vs. 35.8%), whereas at position −175, it achieved higher efficiency (58.9% vs. 54.4%). Importantly, at both sites, the proportion of precisely edited reads, defined as those containing only the intended A-to-G conversion without bystander edits, was substantially higher with ABE8e^Y149V^, exceeding that of ABE8e by 5.3-fold and 8.1-fold, respectively (Fig. 7B).

To further evaluate its therapeutic potential, we tested ABE8e^Y149V^ at four pathogenic SNPs associated with genetic diseases: *POLG* (linked to mitochondrial DNA depletion syndromes) (Zhao et al., 2024), *SCN1A* (Dravet syndrome) (Zhao et al., 2024), *MYH7* (hypertrophic cardiomyopathy) (Reichart et al., 2023), and *COL7A1* (recessive dystrophic epidermolysis bullosa) (Banskota et al., 2022). We integrated 158-bp genomic segments containing these mutations into the HEK293T genome to mimic endogenous contexts. ABE8e^Y149V^ showed enhanced editing efficiency over ABE8e at the *POLG* and *SCN1A* loci, showed similar activity at *MYH7*, and slightly reduced efficiency at *COL7A1*. However, at all four loci, ABE8e^Y149V^ consistently yielded a significantly higher proportion of perfectly edited reads compared with ABE8e (*POLG*: 77.7% vs. 39.8%; *SCN1A*: 26.7% vs. 4.7%; *MYH7*: 74.4% vs. 62.5%; *COL7A1*: 78.1% vs. 67.9%) (Fig. 7C–F).

These results demonstrate that ABE8e^Y149V^ enables efficient and highly precise correction of disease-associated mutations, with a markedly reduced incidence of bystander editing.

### ABE8e^Y149V^ editing of *Hpd* rescues lethality in HTI mice

**Figure 8.**
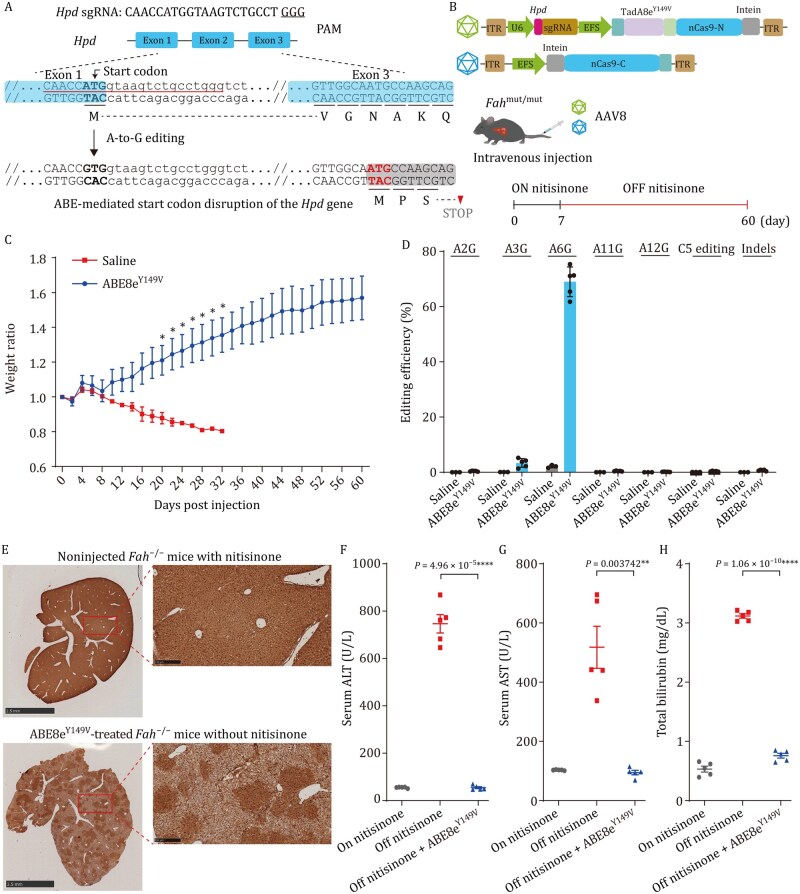
**ABE8e^Y149V^ rescued the lethal phenotype of hereditary tyrosinemia type I in mice**. (A) Scheme of the ABE8e^Y149V^-mediated gene disruption strategy for the mouse *Hpd* gene. The *Hpd* sgRNA was designed to target the start codon (ATG) of the *Hpd* gene, positioning the targeted A at position 6, which is within the editing window of ABE8e^Y149V^. The targeted A in the start codon and PAM sequence are bold and underlined, respectively. The targeted A to G mutation can induce *Hpd* gene disruption. (B) Schematic of the dual-AAV system for *in vivo* delivery of ABE8e^Y149V^ and *Hpd* sgRNA. *Fah^−^*^/^^*−*^ mice were injected with dual AAVs, kept on nitisinone for 7 days, and then weaned off the drug. (C) Body weight ratio in *Fah^−^*^/^^*−*^ mice following treatment with ABE8e^Y149V^-*Hpd* sgRNA versus saline. Weight ratio was calculated as the body weight at each time point divided by the initial weight on day 0 for each individual mouse. Data are presented as mean ± SEM. *P* values for days 20, 22, 24, 26, 28, 30, and 32 were 0.046, 0.042, 0.046, 0.041, 0.039, 0.036, and 0.030, respectively. (D) Deep sequencing analysis of the *Hpd* genomic region of liver DNA from *Fah*^−/−^ mice at day 30 postinjection. Data are presented as mean ± SEM (*n *= 5). (E) Immunohistochemistry (IHC) staining of livers with anti-HPD antibody in noninjected *Fah*^−/−^ mice with nitisinone and in dual AAVs-treated *Fah*^−/−^ mice without nitisinone at day 30 postinjection. (F–H) Serum levels of aspartate transaminase (AST), alanine transaminase (ALT) and total bilirubin for liver damage were assessed in noninjected *Fah*^−/−^ mice with nitisinone, saline-injected *Fah*^−/−^ mice without nitisinone at day 30 postinjection, and dual AAVs-treated *Fah^−^*^/^^*−*^ mice without nitisinone at day 30 postinjection. Data are presented as mean ± SEM (*n *= 5). *P* values were calculated by two-sided unpaired *t*-test. *P *< 0.05 was considered significant. *P < 0.05. **P < 0.01. ***P < 0.001. ****P < 0.0001. HPD, hydroxyphenylpyruvate dioxygenase.

Following the efficient correction of disease-associated mutations in human cells, we next evaluated the *in vivo* therapeutic potential of ABE8e^Y149V^ in a mouse model of HTI, a fatal metabolic disorder caused by mutations in the *Fah* gene. HTI results from loss-of-function mutations in fumarylacetoacetate hydrolase (FAH), a key enzyme in the tyrosine degradation pathway. This deficiency leads to the accumulation of toxic tyrosine metabolites, causing hepatocyte damage, progressive liver failure, and weight loss in both humans and mice (Morrow and Tanguay, 2017). Pharmacological inhibition of an upstream enzyme, hydroxyphenylpyruvate dioxygenase (HPD), using nitisinone has been shown to prevent the accumulation of these toxic intermediates and thereby rescue the disease phenotype (Song et al., 2020; Yin et al., 2014). Previous reports have demonstrated that disrupting *Hpd* using either Cas9 nuclease-mediated editing (Pankowicz et al., 2016) or cytosine base editor (CBE)-mediated introduction of a premature stop codon (Rossidis et al., 2018) can restore liver function and survival in this model. Building upon these strategies, we employed a precise base editing approach to disrupt *Hpd* by mutating its start codon *in vivo* using ABE8e^Y149V^ (Fig. 8A), thereby silencing gene expression without introducing double-strand breaks or indels.

Due to the limited cargo capacity of AAV (less than 5 kb), we employed a dual-AAV delivery system for ABE8e^Y149V^. The editor was split into N- and C-terminal fragments, each fused to the Cfa intein (Stevens et al., 2016), enabling posttranslational reconstitution *in vivo* (Fig. 8B). The two constructs were packaged into AAV serotype 8 (AAV8) and delivered by tail vein injection into 6- to 8-week-old *Fah^−/−^* mice. In parallel, we also delivered split ABE8e using a dual-AAV system to enable direct comparison of initial editing efficiencies. In a cohort maintained on nitisinone for 2 weeks postinjection, targeted deep sequencing analysis of liver tissues revealed that ABE8e^Y149V^ and ABE8e achieved comparable average on-target editing efficiencies (35.8% vs. 35.5%), whereas ABE8e^Y149V^ induced markedly lower levels of bystander A-to-G edits, C edits, and indel formation relative to ABE8e, indicating improved editing precision even without selective enrichment (Fig. S9).

In addition, we evaluated another cohort of ABE8e^Y149V^-treated mice and saline-injected *Fah*^−/−^ controls after nitisinone withdrawal. Following AAV injection, mice were maintained on oral nitisinone treatment in drinking water for 7 days, after which nitisinone was withdrawn (Fig. 8B). By day 32 postinjection, the saline-injected control mice lost on the average 20% of their body weight and had to be euthanized. By contrast, mice injected with ABE8e^Y149V^-expressing AAVs were phenotypically indistinguishable from wild-type mice, with normal weight gain in the absence of nitisinone (Fig. 8C). Mice were harvested on day 30 to examine both the editing frequency and liver function. In ABE8e^Y149V^-treated mice, deep sequencing of the *Hpd* genomic region in liver tissues demonstrated the occurrence of precise editing of A-to-G at the A6 position, with an average base-editing efficiency of 69.0% and negligible bystander A-to-G edits, bystander C edits, or indels (Fig. 8D). Additionally, immunohistochemistry (IHC) staining of liver sections with anti-HPD antibody detected widespread patches of HPD-negative hepatocytes (Fig. 8E). By monitoring the levels of serum biomarkers, including aspartate transaminase (AST), alanine transaminase (ALT), and total bilirubin, we found that the liver functions, which were defective in control saline-injected *Fah*^–/–^ mice (without nitisinone), were totally rescued by the ABE8e^Y149V^-treatment of *Fah*^–/–^ mice (without nitisinone), to a level comparable with the wild-type mice and nitisinone-treated *Fah*^–/–^ mice (Fig. 8F–H). These findings demonstrate that ABE8e^Y149V^ enables efficient and precise *in vivo* base editing and effectively rescues the disease phenotype in adult HTI mice, highlighting its strong potential for therapeutic genome editing.

## Discussion

The number of studies investigating potential therapeutic applications of base editors has been rapidly increasing (Chiesa et al., 2023; Naddaf, 2023). However, the off-target and potentially deleterious effects induced by the deaminase of base editors (Fiumara et al., 2024; Pacesa et al., 2024; Yan et al., 2023; Zuo et al., 2019) highlight the urgent need for rigorous evaluation and effective mitigation strategies. A landmark study showed that the CBE “BE3” could induce widespread *de novo* SNVs throughout the mouse genome (Zuo et al., 2019), potentially causing adverse phenotypic effects (Yan et al., 2023). These off-target effects were attributed to nonspecific interactions between the deaminase and exposed single-stranded DNA (ssDNA) regions (Zuo et al., 2019). In this study, we focused on ABE8e, a versatile and highly efficient ABE that is widely used in recent preclinical gene therapy studies in a variety of mouse models of genetic disorders (Arbab et al., 2023; Lebek et al., 2023; Liao et al., 2023; Newby et al., 2021; Reichart et al., 2023; Sun et al., 2024). Using the GOTI method, we found that ABE8e introduces hundreds of genome-wide off-target SNVs in mouse embryos, while its predecessor, ABE7.10, showed negligible off-target effect. These results support the idea that the eight mutations introduced during the evolution of TadA8e from TadA* may have substantially increased its catalytic activity but at the cost of reduced target specificity, likely through enhanced activity on exposed ssDNA during replication or transcription.

To address this limitation, we performed saturation mutagenesis on the eight amino acid residues unique to TadA8e. This systematic screen identified Y149V as a single-residue substitution that significantly reduces both DNA and RNA off-target effects while maintaining high on-target editing activity. Previous structural analysis of ABE8e revealed that Y149 is located at the entrance of the catalytic pocket and forms hydrogen bonds with the nontarget DNA strand (NTS), particularly stabilizing ssDNA near the 3′ end of the editing window (Lapinaite et al., 2020). Substituting this polar tyrosine with a hydrophobic valine likely destabilizes nonspecific interactions at the boundary of the window, thereby narrowing the editing range and reducing promiscuous substrate binding. Although this mechanistic insight helps explain the improved specificity of ABE8e^Y149V^ without compromising central-site editing efficiency, the precise molecular mechanism underlying these effects remains to be fully elucidated. Further structural and dynamic studies of the deaminase-DNA interface will be necessary to clarify how the Y149V substitution modulates enzyme specificity and activity.

We systematically benchmarked ABE8e^Y149V^ against four previously reported high-fidelity ABE8e variants, including ABE8e^V106W^ (Rees et al., 2019; Richter et al., 2020), ABE8e^V82G^ (Grünewald et al., 2019b), ABE8e^K20A/R21A^ (Grünewald et al., 2019b), and ABE9 (Chen et al., 2023). Across both a 102-sgRNA library and 15 endogenous target sites, ABE8e^Y149V^ achieved peak on-target editing efficiencies comparable with ABE8e, similar to ABE8e^V106W^, ABE8e^V82G^, and ABE8e^K20A/R21A^, and significantly higher than ABE9. Notably, among these high-efficiency variants, ABE8e^Y149V^ markedly reduced genome-wide and transcriptome-wide off-target SNVs to nearly background levels.

In conclusion, our study identifies ABE8e^Y149V^ as an advanced adenine base editor that combines robust on-target performance with high fidelity, achieving a favorable balance between efficiency and specificity and representing a promising tool for precise and safe therapeutic genome editing.

## Supplementary Material

pwag006_Supplementary_Data

## Data Availability

The raw data were deposited in the National Center for Biotechnology Information (NCBI) Sequence Read Archive database with the accession codes PRJNA1142415.

## Abbreviations

AAV, adeno-associated virus; ABEs, adenine deaminase base editors; BCL11A, B cell chronic lymphocytic leukaemia/lymphoma 11A; COL7A1, collagen type VII alpha 1 chain; Dmd, duchenne muscular dystrophy; Fah, fumarylacetoacetate hydrolase; HBG1/2, paralogous gamma-globin genes; Hpd, hydroxyphenylpyruvate dioxygenase; HT1, hereditary tyrosinemia type I; MYH7, myosin heavy chain gene 7; PAM, protospacer adjacent motif; PCSK9, proprotein convertase subtilisin/kexin type 9; POLG, DNA polymerase subunit gamma; SCN1A, sodium voltage-gated channel alpha subunit 1; SNVs, single nucleotide variants; Tyr, tyrosinase.

## Code availability

All software used in this study is publicly available.
