# Supplementary material for: Therapeutic adenine base editor with minimized off-target effects
Source: Protein Cell. 2026 Feb 5;17(6):543–59. doi: 10.1093/procel/pwag006 (PMC13225742; doi:10.1093/procel/pwag006)
Supplement: pwag006_Supplementary_Data [file pwag006_supplementary_data.zip › pwag006_Supplementary_Data/Supplementary_Information.docx]

**Therapeutic adenine base editor with minimized off-target effects**

Yongsen Sun^1,3^, Nana Yan^1,3^, Hu Feng^1,3^, Hongjiang Lu^1,2,3^, Zhenrui Zuo^1^, Chikai Zhou^1^, Erwei Zuo^1*^

^1^State Key Laboratory of Genome and Multi-omics Technologies，Shenzhen Branch, Guangdong Laboratory of Lingnan Modern Agriculture, Key Laboratory of Gene Editing Technologies (Hainan), Ministry of Agriculture and Rural Affairs, Agricultural Genomics Institute at Shenzhen, Chinese Academy of Agricultural Sciences, Shenzhen, China.

^2^Key Laboratory of Agricultural Animal Genetics, Breeding and Reproduction, Ministry of Education & College of Animal Science and Technology, Huazhong Agricultural University, Wuhan, China.

^3^These authors contributed equally: Yongsen Sun, Nana Yan, Hu Feng, Hongjiang Lu.

*Corresponding author.

Email: zuoerwei@caas.cn (E.Z.)

**This file includes:**

Methods

Figures S1 to S10

**Methods**

**Cloning**

Editor expression cassettes (Cas9, CBEs and ABEs; Table S2) were cloned into pCMV-editor-backbone, and sgRNAs were cloned into sgRNA backbone (Fig. S1C). Gene templates were obtained from Addgene or synthesized in GENEWIZ. PCR was performed using KOD-Plus-Neo high fidelity DNA polymerase (Toyobo, KOD-401). Recombinant plasmids or different ABE8e variants were constructed using NEBuilder HiFi DNA Assembly Master Mix (New England BioLabs). The sgRNA target oligos were synthesized, annealed, and ligated into *Bbs*I or *Bsa*I sites to generate the sgRNA-expressing vectors. Protospacer and PAM sequences for all target sites are listed in Table S3.

**Generation of stable cell line disease models**

To establish disease-relevant stable cell lines, 158-bp genomic fragments harboring G:C-to-A:T mutations associated with human diseases were cloned into a modified lentiviral backbone derived from lentiCRISPR v2 (#52961), resulting in the constructs Lenti-POLG(R232C)-BFP-T2A-Puro, Lenti-SCN1A(V977M)-BFP-T2A-Puro, Lenti-Myh7(R403Q)-BFP-T2A-Puro, and Lenti-COL7A1(R185X)-BFP-T2A-Puro. Lentiviral particles were produced by transfecting HEK293T cells with the transfer plasmid together with pMD2.G and psPAX2, using polyethyleneimine (PEI, Polysciences) as the transfection reagent. After 48 hours, the viral supernatant was harvested and filtered through a 0.45-μm low protein-binding membrane (Millipore). HEK293T cells seeded in 12-well plates were transduced with 50 μl of the filtered viral supernatant. Following 24 hours of incubation, cells were subjected to puromycin selection (3 μg ml⁻¹) for 72 hours to enrich for successfully transduced cells. Cells confirmed to carry a single-copy integration were expanded and continuously cultured for future transfections.

**Cell culture, transfection, FACS, and genomic DNA extraction**

HEK293T and 102-sgRNA cells were cultured in Dulbecco’s modified Eagle medium (DMEM, Gibco) supplemented with 10% Fetal Bovine Serum (FBS; Biological Industries) and 1% penicillin/streptomycin (Beyotime) at 37 °C in a 5% CO_2_ incubator. For parallel comparison of editing efficiency, editor plasmids were either transfected into 102-sgRNA cells alone or co-transfected with sgRNA-expressing vectors into HEK293T cells using PEI, following the manufacturer’s protocols. Forty-eight hours after transfection, double-positive cells expressing the GFP and mCherry were isolated by fluorescence-activated cell sorting (FACS, BD FACSAria III). The gating strategy used to identify GFP^+^ and mCherry^+^ cells is provided in Fig. S10. Approximately, 500,000 102-sgRNA cells were sorted, and genomic DNAs (gDNAs) were extracted using the TIANamp Genomic Kit (Tiangen). Additionally, 20,000 HEK293T cells were sorted and gDNAs were extracted using the One-Step Mouse Genotyping Kit (Vazyme).

**Targeted deep sequencing**

The genome sequences of targeted sites were amplified with nested PCR using Takara Ex Taq Polymerase (Takara). Barcodes were added to distinguish samples in the second round of PCR. The PCR products were purified using a universal DNA purification kit (TIANGEN) and sequenced by 150-bp paired-end reads on the Illumina NovaSeq 6000 platform (Genewiz Co. Ltd). The raw data from deep-sequencing were demultiplexed using fastq-multx ^49^, and the reads for each sample were aligned to the reference target sequence by CRISPResso2 (v2.0.32) ^50^. On-target editing efficiency for each target site was calculated using in-house scripts (v5.26.2).

**Animal care**

Heterozygous Ai9 (B6.Cg-Gt (ROSA) 26Sortm9 (CAG-td-Tomato) Hze/J; JAX strain 007909) male mice were mated with female C57BL/6 mice (4 weeks old) for embryo collection. ICR female mice were used as recipients. *Fah^-/-^* mice were used for *in vivo* editing experiments. The use and care of animals complied with the guidelines of the Life Sciences Ethics Committee of Agricultural Genomics Institute, Chinese Academy of Agricultural Sciences.

***In vitro* transcription of mRNA and sgRNA**

For Cas9 and base editor mRNAs, T7 promoter was added to the N-terminal of coding region by PCR amplification of the plasmid using primers IVT F and R (Table S4). The purified PCR product was used as the template for *in vitro* transcription (IVT) using mMESSAGE mMACHINE T7 ULTRA kit (Life Technologies). For sgRNA IVT, T7 promoter was added to sgRNA template by PCR amplification of pX330, and the purified PCR product was used as the template for IVT using MEGA shortscript T7 kit (Life Technologies). The editor mRNAs and sgRNAs were purified using MEGA clear kit (Life Technologies).

**Two-cell embryo injection, embryo transplantation and FACS for GOTI**

Four-week-old superovulated C57BL/6 females were mated with homozygous Ai9 males to obtain fertilized embryos from oviducts 24 hours after hCG injection. For the 2-cell embryo injection, a mixture of Cas9 or base editor mRNA (50 ng/μl), sgRNA (50 ng/μl), and Cre mRNA (2 ng/μl) was injected into the cytoplasm of one blastomere of 2-cell embryo 48 hours post hCG injection. The injection was performed in a droplet of M2 medium containing 5 μg/ml cytochalasin B (CB) using a FemtoJet microinjector (Eppendorf) with constant flow settings. Following injection, embryos were cultured in KSOM medium with amino acids at 37 °C under 5% CO2 for 2 hours and then transferred into oviducts of pseudopregnant ICR females at 0.5 dpc.

At embryo day 14.5 (E14.5), embryo tissues were cut into small pieces and digested in 5 mL of 0.05% Trypsin-EDTA (Gibco) at 37 °C for 30 min. The digestion was stopped by adding 5 mL of DMEM medium supplied with 10% FBS. Subsequently, fetal tissues were homogenized using a 1 mL pipette tip, followed by centrifugation of the cell suspension at 200 g for 6 min. The resulting pellet was resuspended in 2 mL DMEM supplemented with 10% FBS. Finally, the cell suspension was filtered through a 40 μm cell strainer to isolate tdTomato^+^ and tdTomato^−^ cells by FACS (Fig. S10).

**Whole genome sequencing (WGS) and data analysis**

The gDNAs of sorted tdTomato^+^ and tdTomato^−^ cells were extracted using the DNeasy blood and tissue kit (Qiagen). WGS was performed with an average coverage of 50× using the BGI DNBSEQ-T7 platform. The qualified sequencing reads were aligned to the reference genome (GRCm39) using BWA (v0.7.17). Picard tools (v2.25.7) were utilized to sort the aligned reads and mark duplicates of the mapped BAM files. Subsequently, genome-wide *de novo* SNVs were called using three algorithms, Mutect2 (v3.5), Lofreq (v2.1.2) and Strelka (v2.7.1) with default parameters, separately. In parallel, the genome-wide *de novo* indels were detected using Mutect2 (v3.5), Scalpel (v0.5.3) and Strelka (v2.7.1) with default parameters. The overlap of three algorithms of SNVs or indels were considered as the true variants.

**RNA libraries preparation, sequencing** **and data analysis**

HEK293T cells were transfected with base editor and sgRNA plasmids, or with GFP and mCherry plasmids as controls. After 48 hours, around 500,000 cells (top 5% GFP/mCherry signal) were collected (Fig. S10) for RNA extraction using Trizol (Ambion). For library construction, mRNAs were first fragmented and converted to cDNA using random hexamers or oligo(dT) primers. Adapters were subsequently ligated to the 5’ and 3’ ends of the cDNA fragments, followed by enrichment and PCR amplification of correctly ligated fragments. Sequencing was performed on the Illumina Novaseq 6000 platform.

For RNA-seq data analysis, initial quality control involved using FastQC (v0.11.3). Subsequently, STAR (v2.7.1) was used in 2-pass mode with default parameters to align the processed reads to the GRCh38 reference genome. Following alignment, Picard tools (v2.25.5) were utilized to sort the aligned reads and identify duplicates in the resulting BAM files. Variants were called using HaplotypeCaller from GATK (v4.2.0.0).

**The AAVs cloning, production and *in vivo* injection**

The AAV vectors were constructed by cloning the U6-*Hpd* sgRNA-N-TadA8e^Y149V^-nCas9 (2–573)-intein and C-intein-nCas9 (574–1368) sequences between AAV serotype 2 ITRs. These vectors, along with helper plasmids and AAV8 packaging plasmids, were transfected into HEK293T cells using PEI. Viral particles were harvested from both the cell culture medium and cells 3–5 days post-transfection. The harvested particles were then subjected to a purification and concentration process as described previously ^47^.

Six- to eight-week-old *Fah^-/-^* mice were intravenously injected with 5 × 10^11^ vector genomes per kilogram of body weight (vg/kg) of each of the dual AAVs in 200 μl saline per mouse via tail vein. Control six- to eight-week-old *Fah^-/-^* mice were injected with 200 μl saline alone.

**Mice weighting, blood collection and serum analysis**

The injected mice were provided with 10 mg/L nitisinone (Sigma-Aldrich) in drinking water for the first 7 days post-injection, after which the nitisinone treatment was discontinued. The body weights of the mice were recorded every other day. Control mice removed from the nitisinone treatment were euthanized upon reaching >20% weight loss. Blood was collected by retroorbital bleed, allowed to clot for 2 hours at room temperature, and then centrifuged at 3,000 g for 15 minutes at 4 °C. Fresh serum samples were used to measured aspartate transaminase (AST), alanine transaminase (ALT) and total bilirubin.

**Immunohistochemistry staining**

Liver tissue was fixed using 4% paraformaldehyde (PFA) at 4 °C for 2 hours, dehydrated with ethanol and embedded in paraffin. Paraffin sections were 5 μm thick, deparaffinized using xylene, and rehydrated with ethanol and distilled water. Anti-HPD antibody (ab133515, Abcam) was used as the primary antibody. Sections were sealed by glass coverslips and photographed using bright-field microscope photograph system (Leica Microsystems).


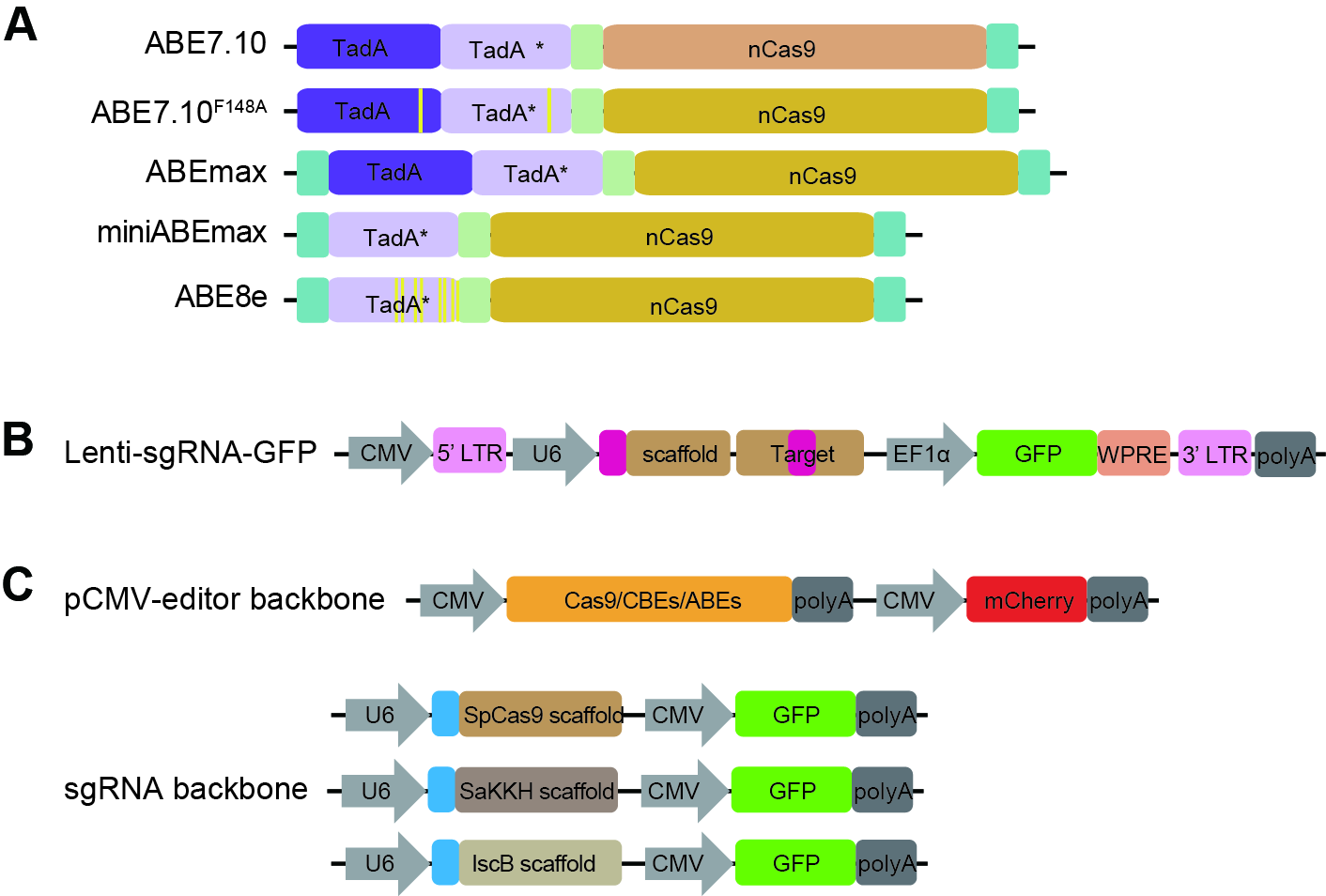


**Figure S1****.** **Schematics of genome editing plasmids (A), lentiviral vector for 102 sgRNAs (B) and vector backbones for editor and sgRNA (C)**.


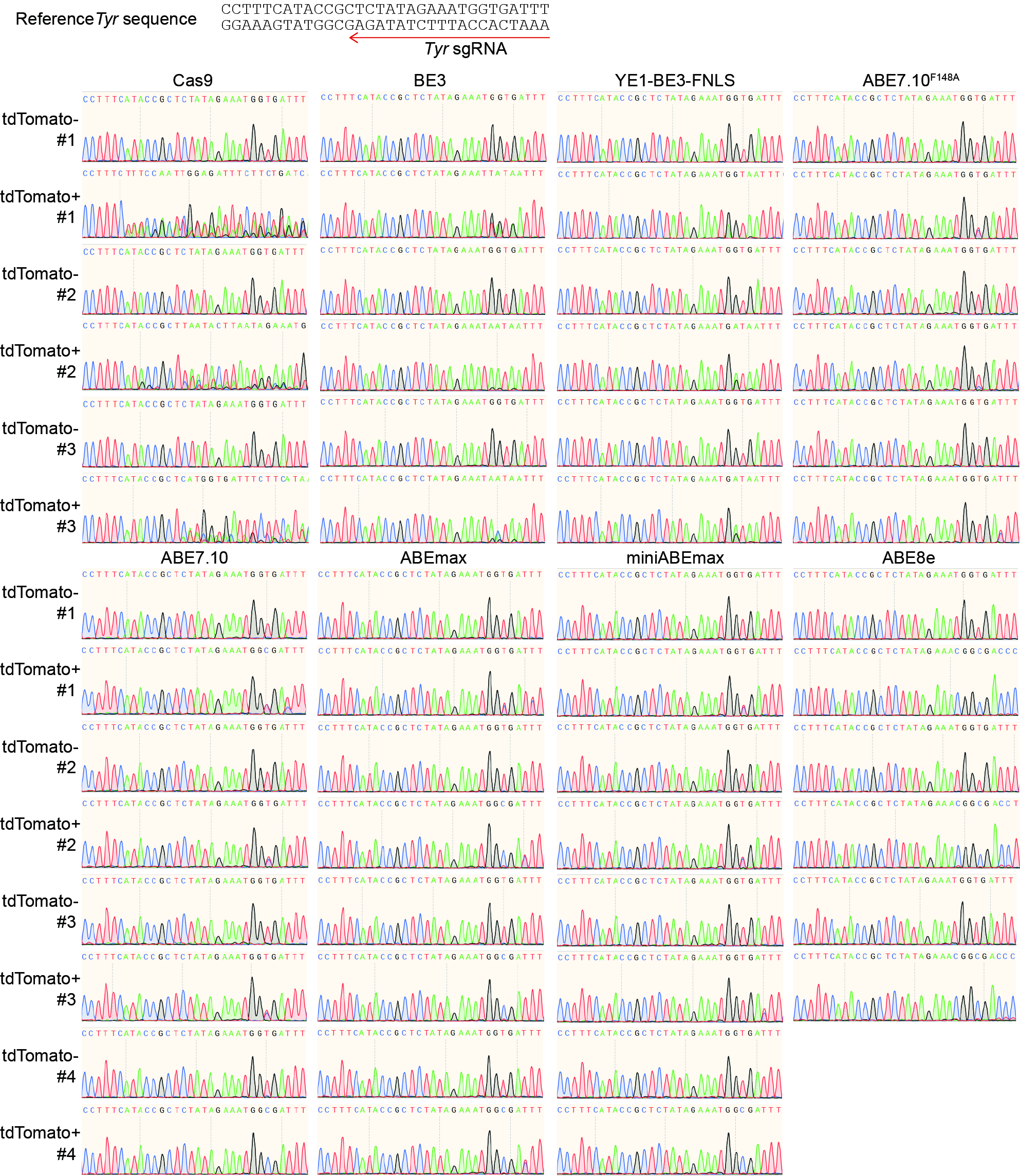


**Figure S2.** **Sanger sequencing showing the editing outcomes at *Tyr* loci in tdTomato^+^ and tdTomato^-^ cells.**


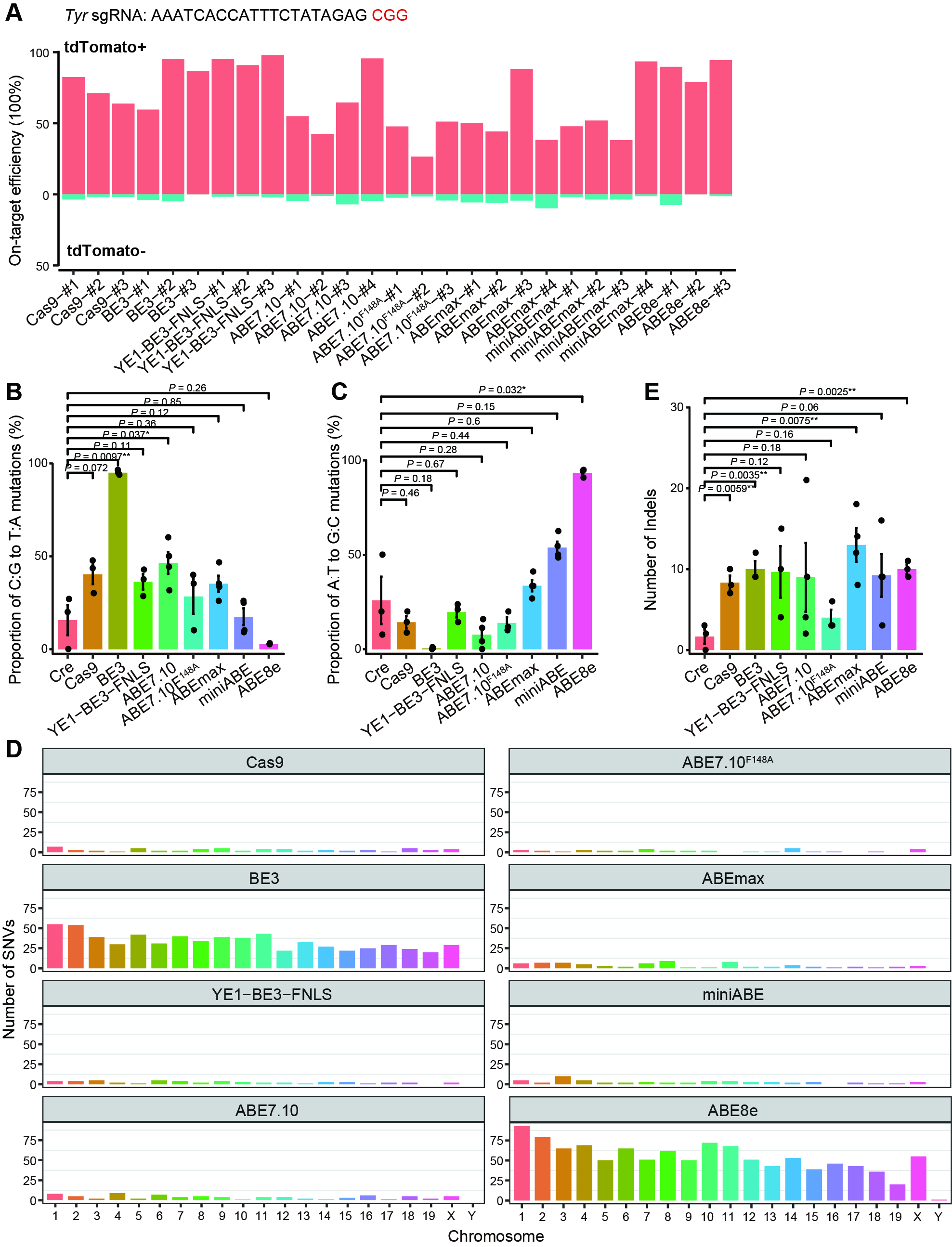


**Figure S3.** **Unbiased genome-wide off-target analysis of Cas9, BE3, YE1-BE3-FNLS, ABE7.10, ABE7.10^F148A^, ABEmax, miniABEmax, ABE8e with *Tyr* sgRNA.**

(A) On-target efficiencies of tdTomato^+^ and tdTomato^−^ cell at *Tyr* locus on the basis of WGS for the treated groups.

(B, C) Proportion of C:G to T:A mutations (B) and A:T to G:C mutations (C) in detected off-target DNA SNVs.

(D) Distributions of off-target DNA SNVs on chromosomes in mouse embryos.

(E) Comparison of the detected off-target indels.

Data are presented as mean ± SEM of biological replicates (n ≥ 3). *P* values were calculated by two-sided unpaired *t*-test. *P* < 0.05 was considered significant. **P* < 0.05; ***P* < 0.01; ****P* < 0.001; *****P* < 0.0001.


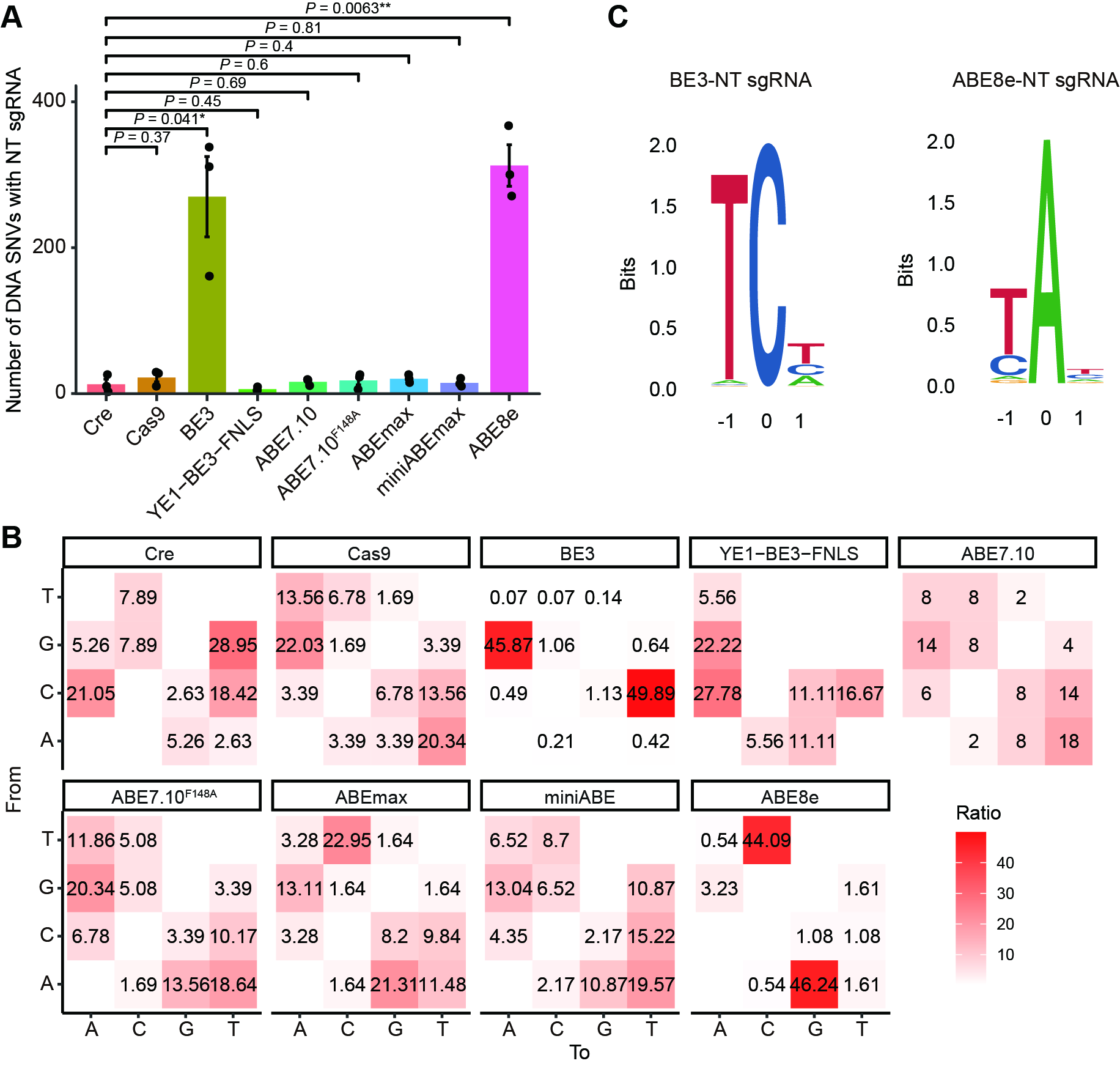


**Figure S4.** **Unbiased genome-wide off-target analysis of Cas9, BE3, YE1-BE3-FNLS, ABE7.10, ABE7.10^F148A^, ABEmax, miniABEmax and ABE8e with non-targeting (NT) sgRNA.**

(A) Comparison of the detected off-target SNVs of Cas9, BE3, YE1-BE3-FNLS, ABE7.10, ABE7.10^F148A^, ABEmax, miniABEmax and ABE8e with identical NT sgRNA.

(B) Distribution of mutation types for groups injected with Cre, Cas9, BE3, YE1-BE3-FNLS, and five ABEs with identical NT sgRNA. The number indicates the percentage of a certain type of SNVs among all SNVs.

(C) Sequence logos derived from off-target DNA SNVs in BE3 and ABE8e groups, respectively.

Data are presented as mean ± SEM of biological replicates (n ≥ 3, as indicated). *P* values were calculated by two-sided unpaired *t*-test. *P* < 0.05 was considered significant. **P* < 0.05; ***P* < 0.01; ****P* < 0.001; *****P* < 0.0001.


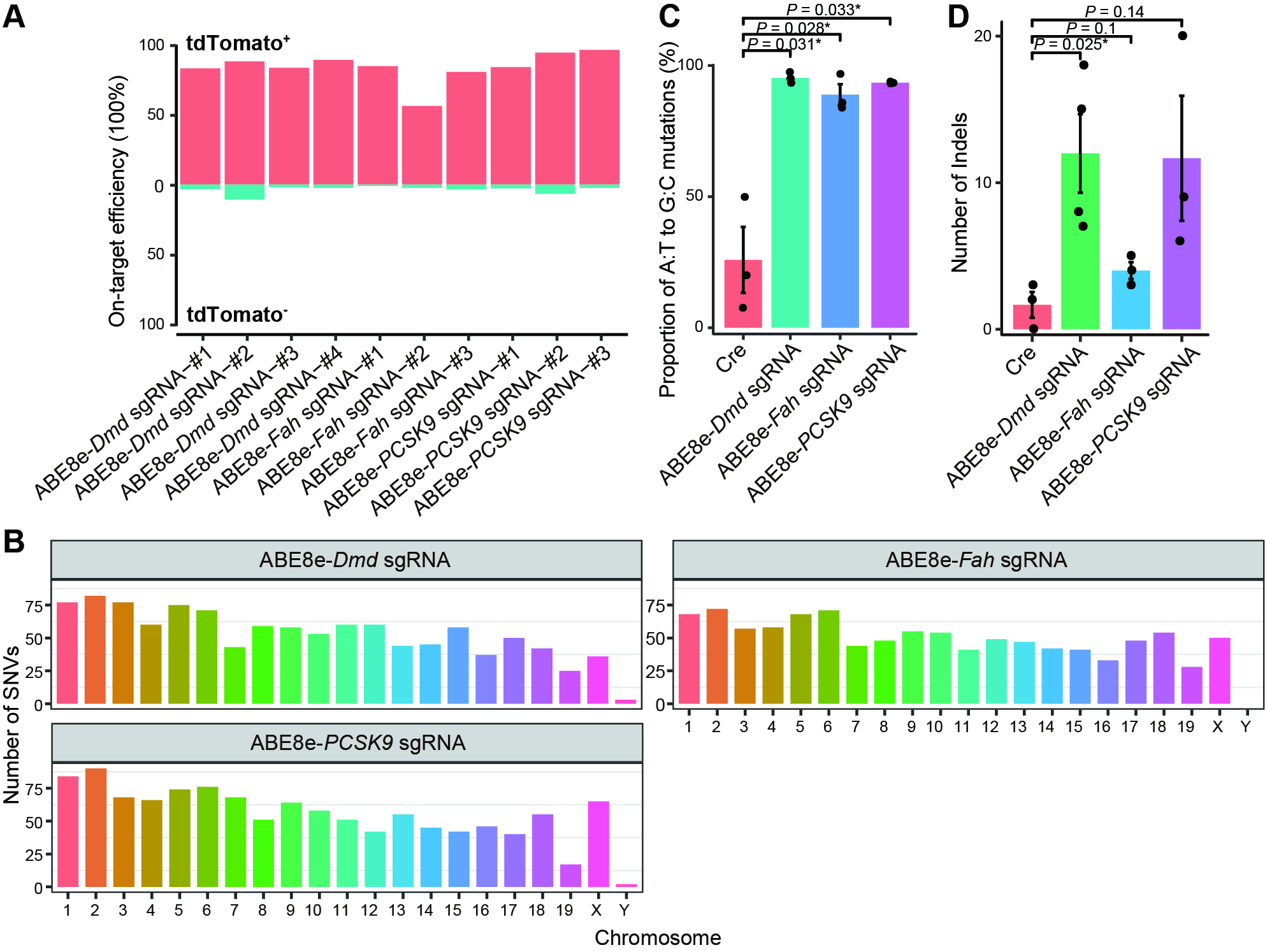


**Figure S5.** **Unbiased genome-wide off-target analysis of ABE8e with *Dmd*, *Fah*, and *PCSK9* sgRNAs.**

(A) On-target editing efficiencies of tdTomato^+^ and tdTomato^−^ cell at *Dmd*, *Fah*, *PCSK9* loci on the basis of WGS for the treated groups.

(B) Distributions of off-target DNA SNVs on chromosomes in mouse embryos.

(C) Proportion of A:T to G:C mutations in detected off-target DNA SNVs.

(D) Comparison of the detected off-target indels.

Data are presented as mean ± SEM of biological replicates (n ≥ 3, as indicated). *P* values were calculated by two-sided unpaired *t*-test. *P* < 0.05 was considered significant. **P* < 0.05; ***P* < 0.01; ****P* < 0.001; *****P* < 0.0001.


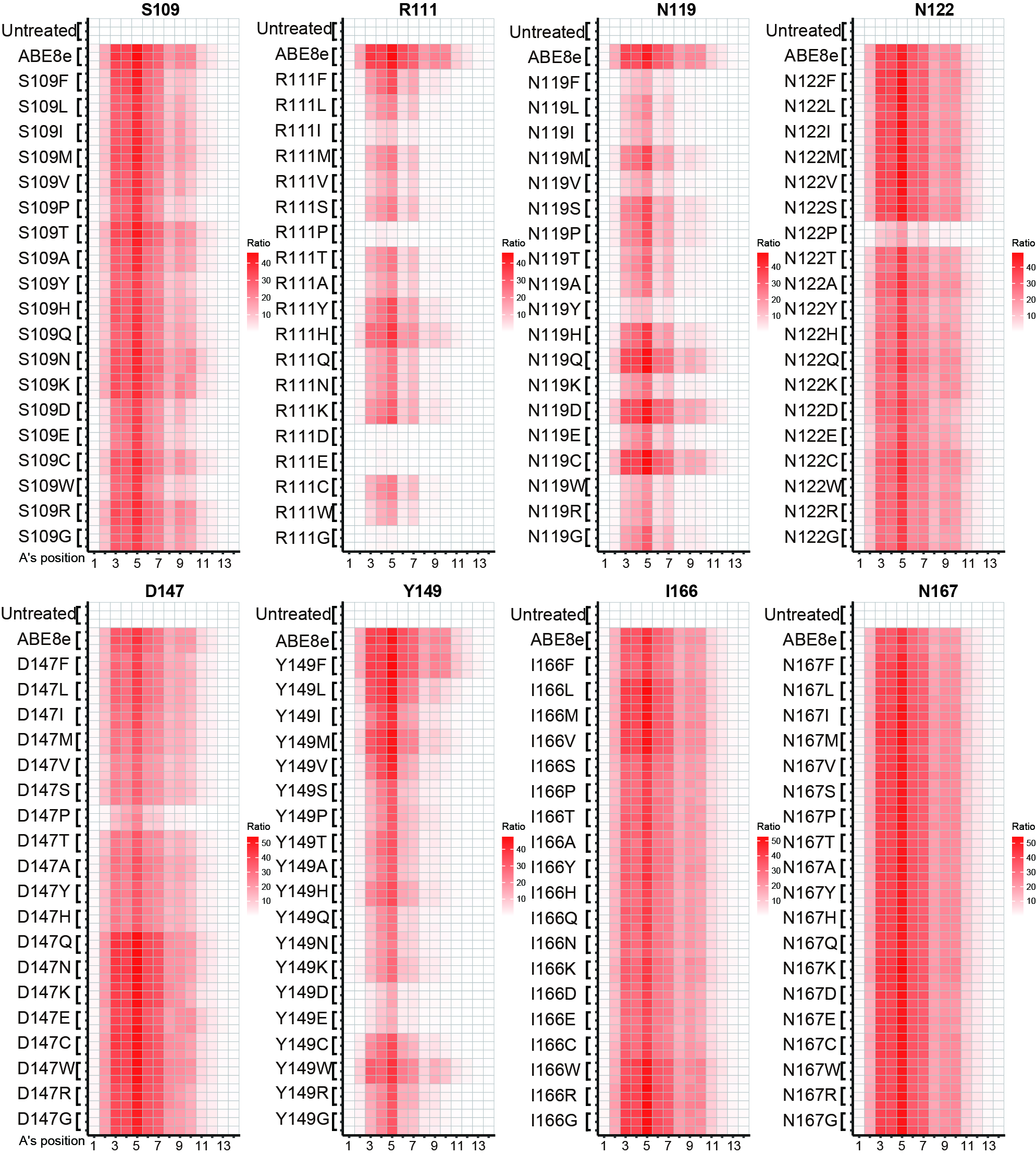


**Figure S6.** **Heat map illustrating the average A-to-G editing efficiencies across positions 1-14 for 152 ABE8e variants generated through saturation mutagenesis at sites S109, R111, N119, N122, D147, Y149, I166, N167**.


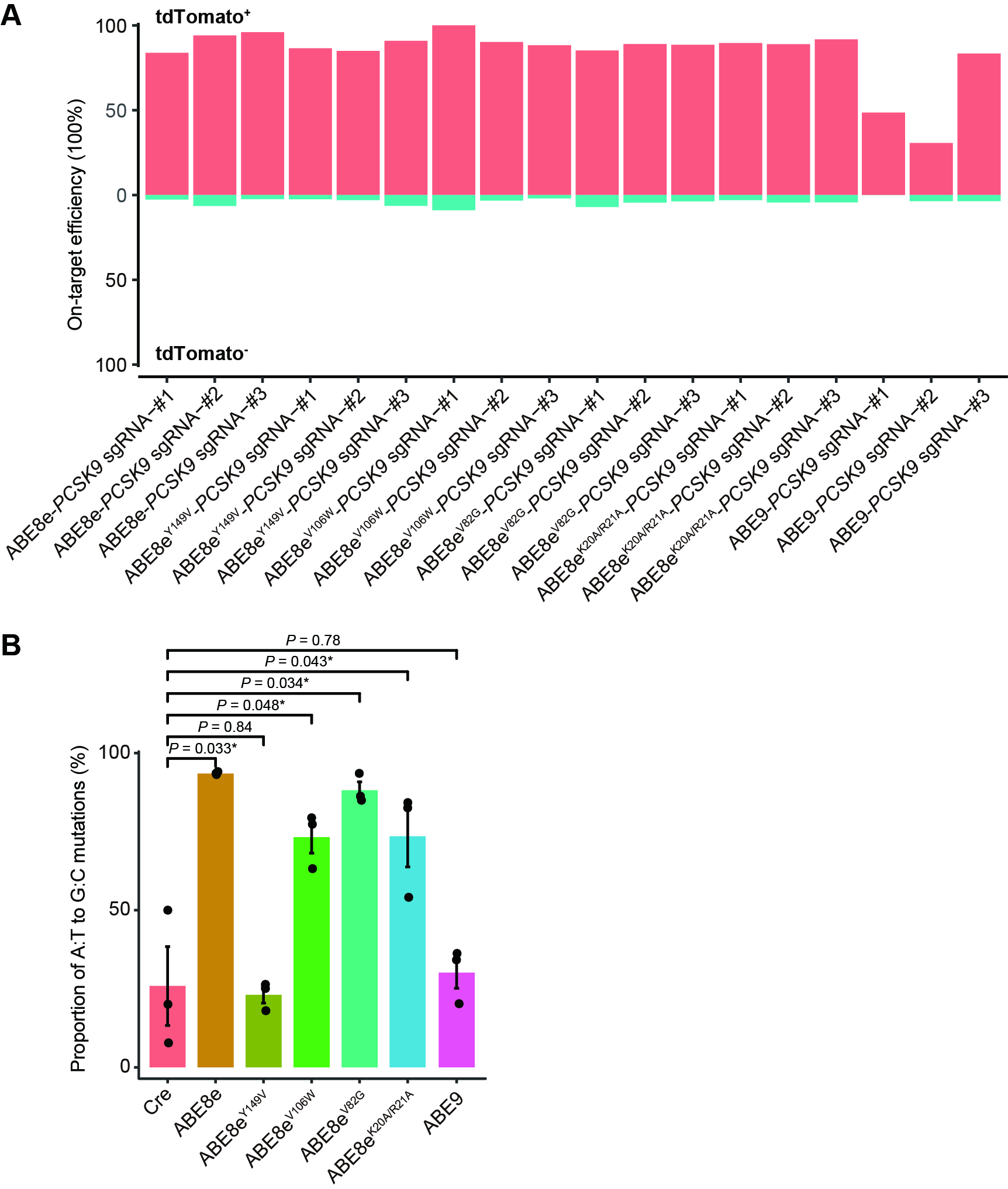


**Figure S7.** **Genome-wide analysis of ABE8e, ABE8e^Y149V^ and other ABE8e variants.** (A) On-target efficiencies of tdTomato^+^ and tdTomato^−^ cell at *PCSK9* locus on the basis of WGS.

(B) Proportion of A:T to G:C mutations in detected off-target DNA SNVs.

Data are presented as mean ± SEM of biological replicates (n ≥ 3, as indicated). *P* values were calculated by two-sided unpaired *t*-test. *P* < 0.05 was considered significant. **P* < 0.05; ***P* < 0.01; ****P* < 0.001; *****P* < 0.0001.


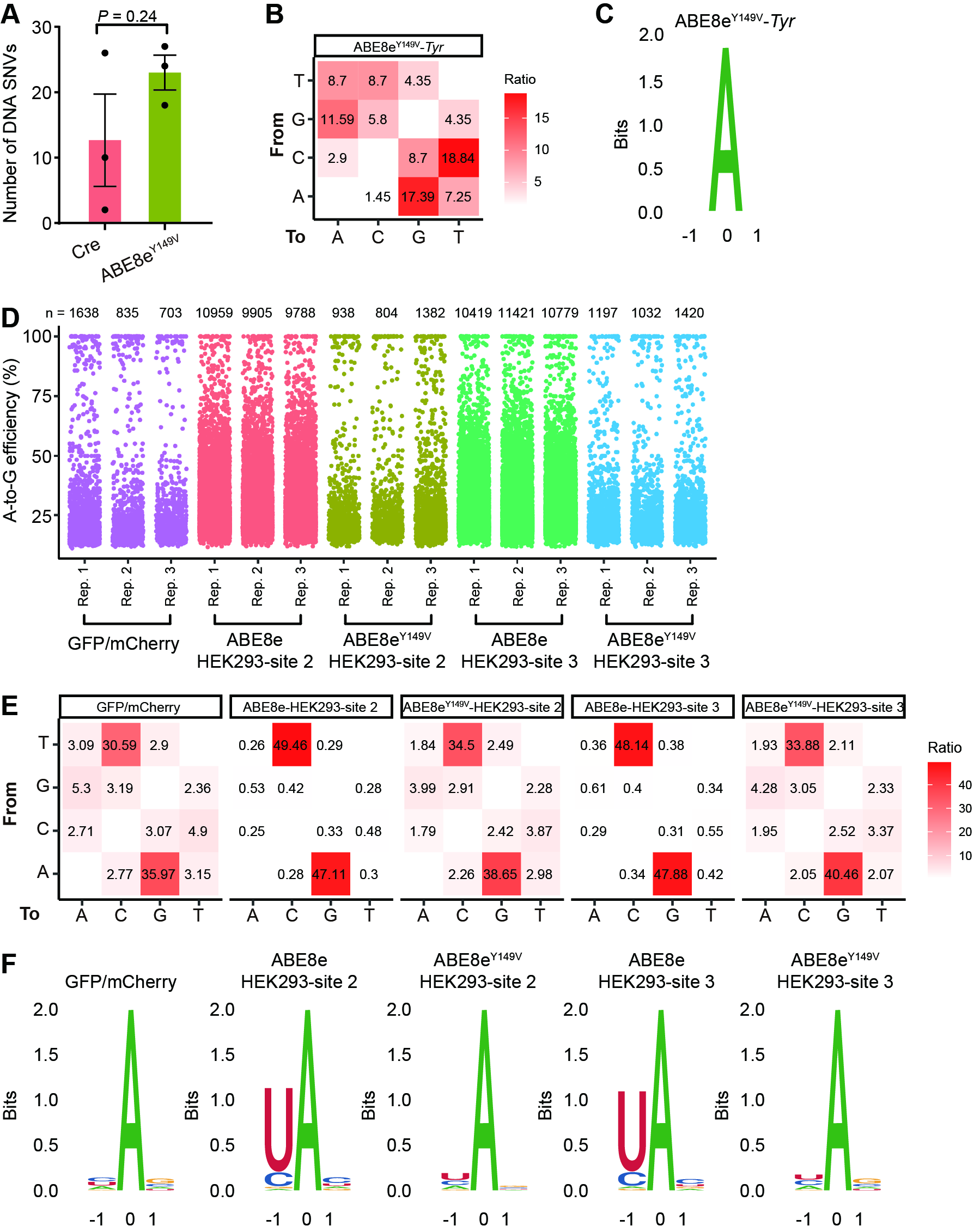


**Figure S8.** **Genome-wide and transcriptome-wide off-target analysis of ABE8e^Y149V^ at additional target sites.**

(A) Comparison of the detected off-target SNVs of ABE8e^Y149V^ at *Tyr* locus with Cre only control.

(B) Distribution of mutation types for groups injected with Cre and ABE8e^Y149V^. The number indicates the percentage of a certain type of SNVs among all SNVs.

(C) Sequence logos derived from off-target DNA SNVs in ABE8e^Y149V^ group.

(D) Jitter plots showing transcriptome-wide RNA A-to-G edits observed in groups including a GFP/mCherry negative control, ABE8e and ABE8e^Y149V^. Each dot represents an individual edited adenine. All experiments (except for the GFP/mCherry control) were performed with co-expression of sgRNA targeting HEK293-site 2 or HEK293-site 3, and in all experiments the cells were sorted for the top 5% of GFP and mCherry signals.

(E) Distribution of RNA mutation types in GFP/mCherry, ABE8e-HEK293-site 2, ABE8e^Y149V^-HEK293-site 2, ABE8e-HEK293-site 3 and ABE8e^Y149V^-HEK293-site 3 groups.

(F) Sequence logos derived from off-target RNA SNVs.

Data are presented as mean ± SEM of biological replicates (n ≥ 3, as indicated). *P* values were calculated by two-sided unpaired *t*-test. *P* < 0.05 was considered significant. **P* < 0.05; ***P* < 0.01; ****P* < 0.001; *****P* < 0.0001.


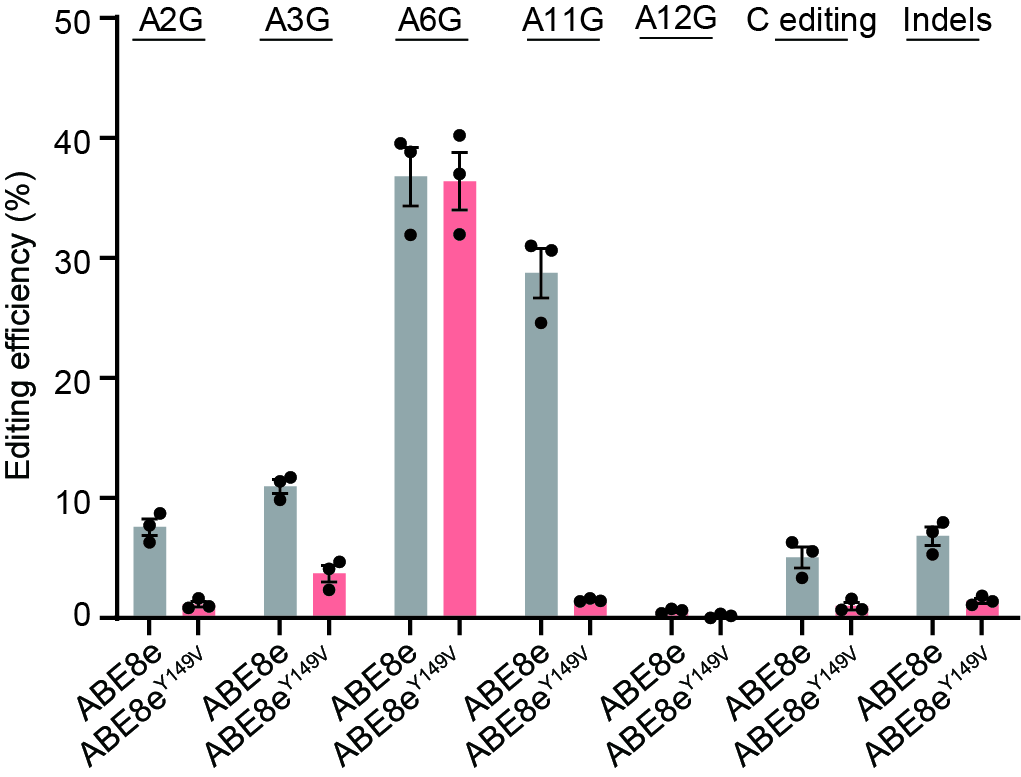


**Figure S9.** **Deep sequencing analysis of the *Hpd* genomic region of liver DNA ABE8e and ABE8e^Y149V^-injected *Fah^-/-^* mice at day 14 post-injection.** Data are presented as mean ± SEM (n = 3).


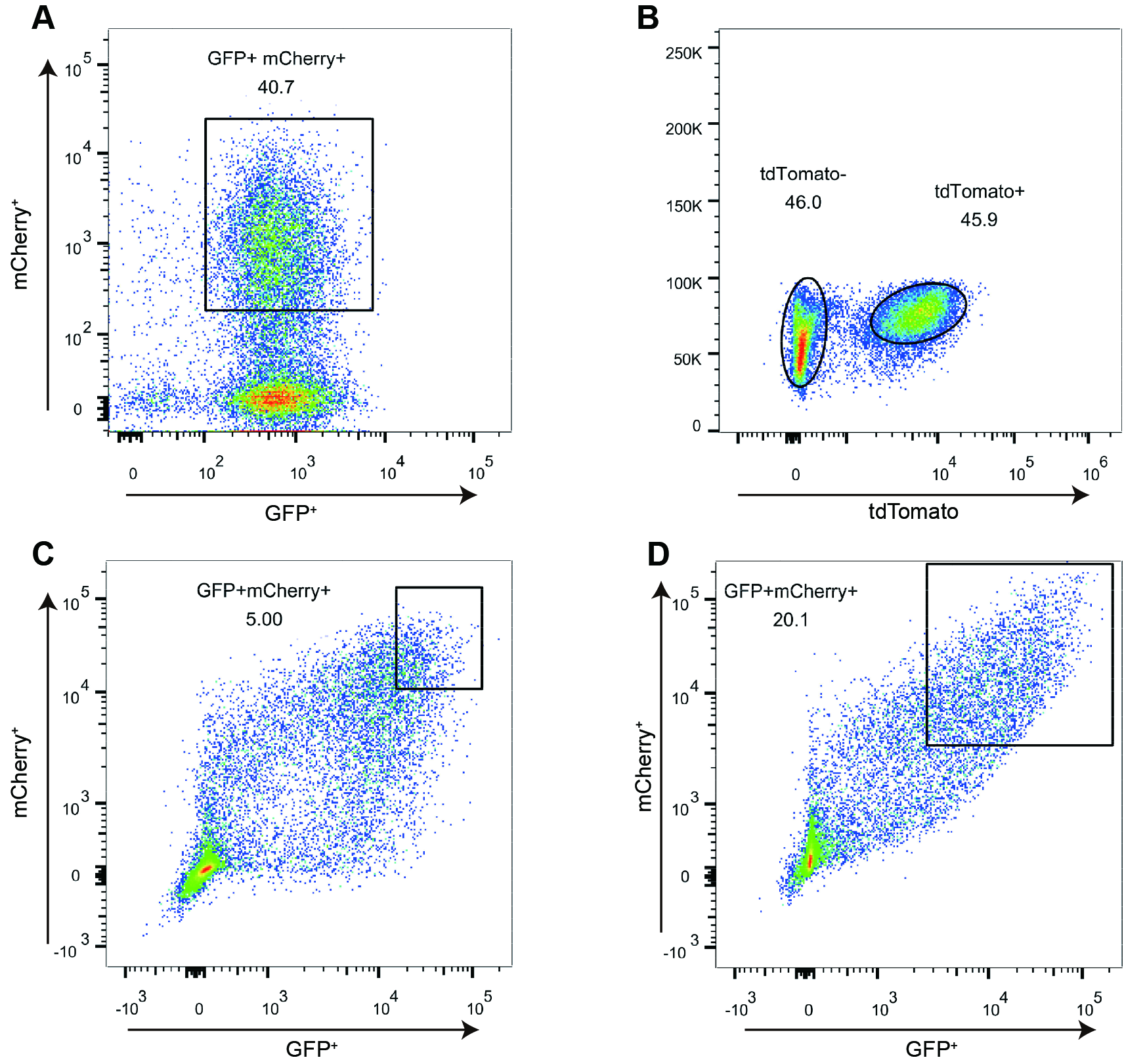


**Figure S10.** **Representative FACS gating strategies for assessing editing efficiencies and off-target effects in multiple experimental systems.**

(A) Sorting GFP^+^ and mCherry^+^ cells to compare the editing efficiencies of gene editing vectors in 102-sgRNA cells.

(B) Sorting tdTomato^+^ and tdTomato^-^ cells to assess the genome-wide off-targets in mouse embryos.

(C) Sorting GFP^+^ and mCherry^+^ cells to evaluate the RNA off-targets in HEK293T cells.

(D) Sorting GFP^+^ and mCherry^+^ cells to analyze the editing efficiencies at endogenous sites in HEK293T cells.
